# Cytotoxic and Luminescent Properties of Novel Organotin Complexes with Chelating Antioxidant Ligand

**DOI:** 10.3390/molecules27238359

**Published:** 2022-11-30

**Authors:** Evgeny Nikitin, Ekaterina Mironova, Dmitry Shpakovsky, Yulia Gracheva, Daniil Koshelev, Valentina Utochnikova, Konstantin Lyssenko, Yury Oprunenko, Dmitry Yakovlev, Roman Litvinov, Mariya Seryogina, Alexander Spasov, Elena Milaeva

**Affiliations:** 1 Faculty of Chemistry, Lomonosov Moscow State University, Leninskie Gory 1-3, Moscow 119991, Russia; 2 Department of Pharmacology and Bioinformatics, Volgograd State Medical University, 1 Pavshikh Bortsov Sq., Volgograd 400131, Russia

**Keywords:** organotin, antioxidants, cytotoxicity, luminescence, chelators, 2,6-di-*tert*-butylphenols, hydrazides

## Abstract

A novel polydentate chelating antioxidant ligand and series of organotin complexes on its base were synthesized and characterized by NMR ^1^H, ^13^C, ^119^Sn, IR spectroscopy, X-ray, and elemental analysis. Their antioxidant activity was evaluated in DPPH and NBT-tests, and as lipoxygenase inhibitory activity. It was shown that ligand alone is a radical scavenger, while introducing tin in the structure of the compound significantly decreases its activity. For the ligand alone the ability to strongly suppress the formation of advanced glycation end products (AGEs) was shown, which may be associated with the established antiradical activity. All synthesized compounds appeared to be moderate lipoxygenase inhibitors. The stability of compounds to hydrolysis under different pH was estimated. The ligand undergoes decomposition after about an hour, while organotin complexes on its base demonstrate vast stability, showing signs of decomposition only after 5 h of experimentation. Cytotoxicity of compounds was studied by standard MTT-test, which showed unorthodox results: the ligand itself demonstrated noticeable cytotoxicity while the introduction of organotin moiety either did not affect the toxicity levels or reduced them instead of increasing. Organotin complexes possess luminescence both as powders and DMSO solutions, its quantum yields reaching 67% in DMSO. The combination of luminescence with unique cytotoxic properties allows us to propose the synthesized compounds as perspective theranostic agents.

## 1. Introduction

Oncological diseases are serious medical and social problems [1]. Platinum drugs are a common remedy [2], yet they possess flaws—such as low selectivity, a plethora of side effects, and tumor cell resistance [3]. This marks an urge to design new chemotherapeutic agents based on other exogenic metals. Tin can be the one—long known for the toxicity of its organic derivatives [4,5], it was chosen as a core metal for the drug Rostaporfin [6], and approved FDA for the treatment of age-related macular degeneration (ARMD). Organotin-based compounds are now being tested in vitro and in vivo as prospective candidates for chemotherapy [7,8]. Despite this, their mechanism of cytostatic action is not entirely clear. Tin is known to induce apoptosis by different mechanisms, including caspases activation [9], spindle failure [10], an increase in intracellular Ca^2+^ concentration [9], etc. One of them is oxidative stress induction [11], involving the generations of vast amounts of reactive oxygen species (ROS), highly reactive species, capable of dealing with cellular damage by starting devastating chain radical reactions [12].

The introduction of antioxidants—compounds that scavenge free radicals and help living cells maintain their redox status—may help to overcome the side effects caused by organotin [13]. Indeed, 2,6-Di-*tert*-butylphenols are known for their antioxidant properties [14], and they are used in many fields of modern chemistry as polymerization stabilizers [15], food supplements [16], and drugs as well [17]. The introduction of this pendant into the structure of organotin pharmaceuticals may help to attenuate the toxic impact on various cell types and lower the overall cytostatic impact on the living organism [18].

The core of our approach lies in the fact, that some tumor cells possess acidic pH, on the other hand, the pH of the healthy cells is almost neutral (pH = 7.4). Introducing the cytoprotective antioxidant group helps to attenuate compound toxicity—the radical scavenging properties of the phenolic OH group will be blocked due to the protonation in the acidic medium while remaining intact in the healthy cells. This approach was implemented in earlier research [18,19] and manifested in this study as well. Considering these features allow one to achieve an optimal level of cytotoxicity and selectivity for the potential pharmacological agent.

Drug distribution monitoring may help to better understand its pharmacokinetics [20], improve the treatment quality for potential patients and elucidate the possible mechanism of organotin compounds’ physiological activity. This can be implemented through the theranostics strategy. This novel principle involves a combination of therapy and diagnostics, dictating specific demands to molecule properties [21]. If designed correctly, the theranostic drug may not only cure specific diseases, but may complete some diagnostic tasks, saving time, money, and, probably, patients’ lives. Despite being initially predominantly radiopharmaceuticals [22], their action principle has drastically widened nowadays. Luminescent drugs possess high sensitivity and resolution and fit theranostic strategy just fine, they can be used for bioimaging of different physiological systems [23,24,25].

The present study was aimed at the synthesis of luminescent organotin complexes bearing antioxidant phenolic pendant and in vitro screening of their antioxidant and biological activity. Radical scavenging potency was monitored by DPPH and NBT-tests, antiglycation activity, and lipoxygenase inhibitory assessment was carried out. Biological activity was monitored as a regular MTT-test on four different cell lines, and luminescent properties were evaluated.

## 2. Results and Discussion

### 2.1. Synthesis

**Scheme 1:**
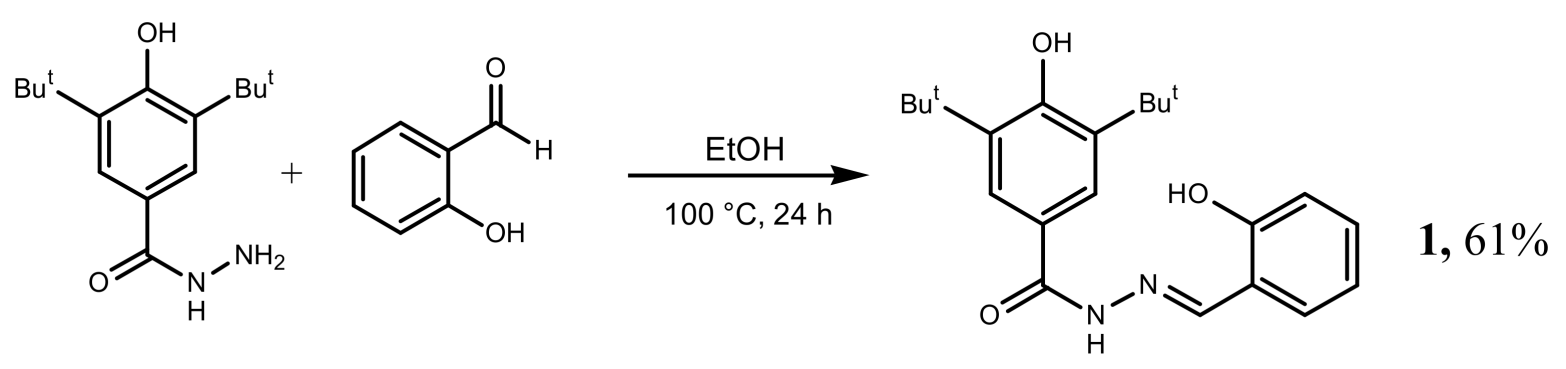
Synthesis of the ligand **1**.

Synthesis of polydentate ligand **1** was carried out as a standard procedure of Schiff bases obtaining, i.e.—vigorous refluxing of solution of 3,5-di-*tert*-butyl-4-hydroxybenzoic hydrazide and salicylic aldehyde in EtOH (Scheme 1) followed by in vacuo solvent removing and rinsing with water. The product was then dried in air for 24 h to give colorless powder. 3,5-Di-*tert*-butyl-4-hydroxybenzoic hydrazide was obtained according to a known method [26].

**Scheme 2:**
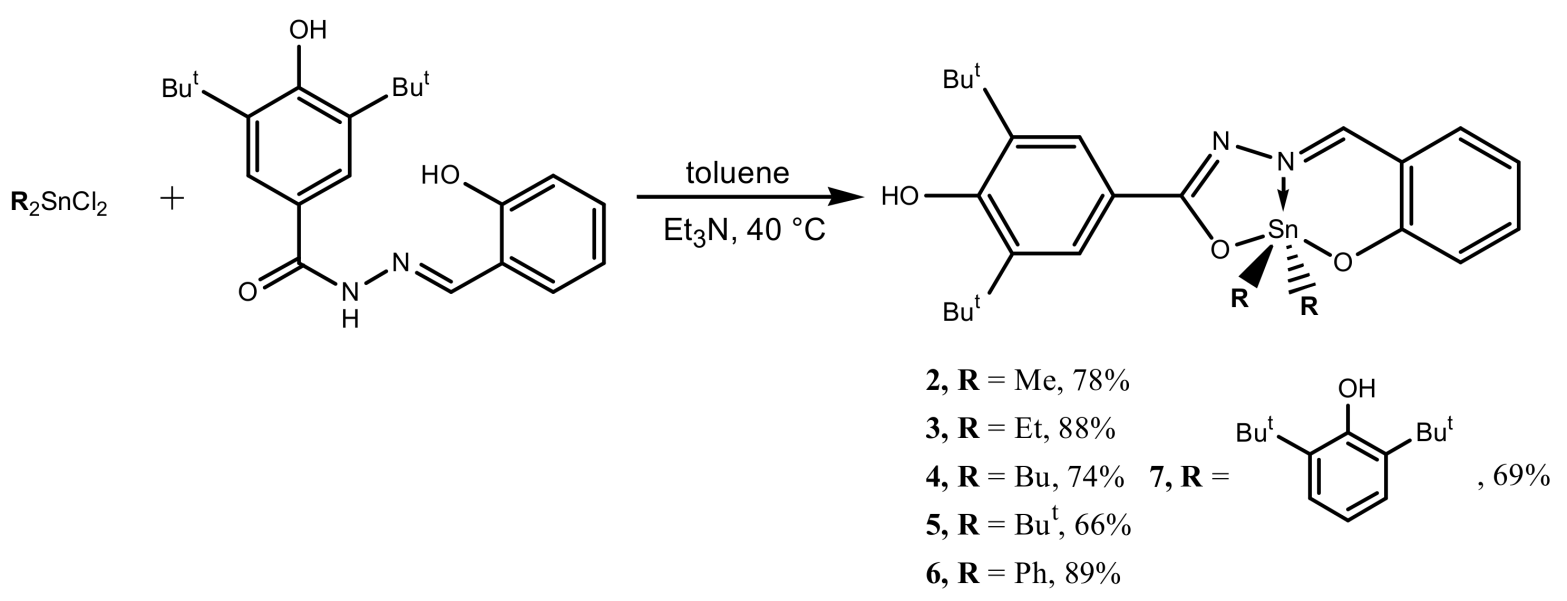
Synthesis of complexes **2**–**7**.

Synthesis of complexes **2**–**7** was carried out by gentle heating of the solution of ligand **1** and corresponding organotin precursor R_2_SnCl_2_ in the presence of triethylamine as a base (Scheme 2). This was followed by in vacuo solvent removing, dissolving crude product in Et_2_O, filtering off Et_3_N·HCl, and subsequently removing Et_2_O in vacuo. Complexes **2**–**7** appeared as vivid yellow-green powders with yields of 66–89%, with slight luminescence under the daylight.

**Scheme 3:**
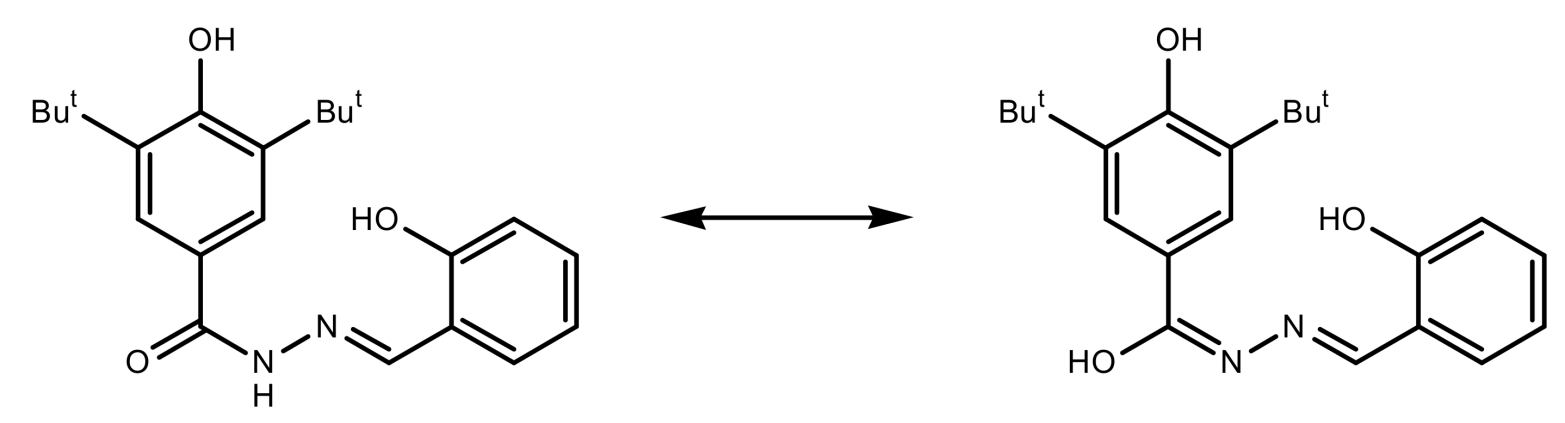
Amide-iminol tautomeric transition of ligand **1** prior to coordination to tin.

Compounds **1**–**7** are stable in air and in solutions in the majority of solvents except for CH_3_Cl, which causes rapid decomposition of ligand **1**. The synthesized series was characterized by NMR ^1^H, ^13^C, ^119^Sn, IR spectroscopy, X-ray, and elemental analysis. The data obtained by ^1^H NMR showed the disappearance of salicylic OH proton and amidic NH proton as well as the Schiff proton shift in complexes **2**–**7**, which signals about ligand **1** undergoing amide-iminol tautomeric transition (Scheme 3) prior to coordination to a tin atom which was shown in the previous study [27] and proven by X-ray analysis. This type of coordination involves the formation of a polycyclic conjugated system C=N-N=C with tin atom and it leads to increased stability of compounds.

#### Molecular Structure

**Figure 1:**
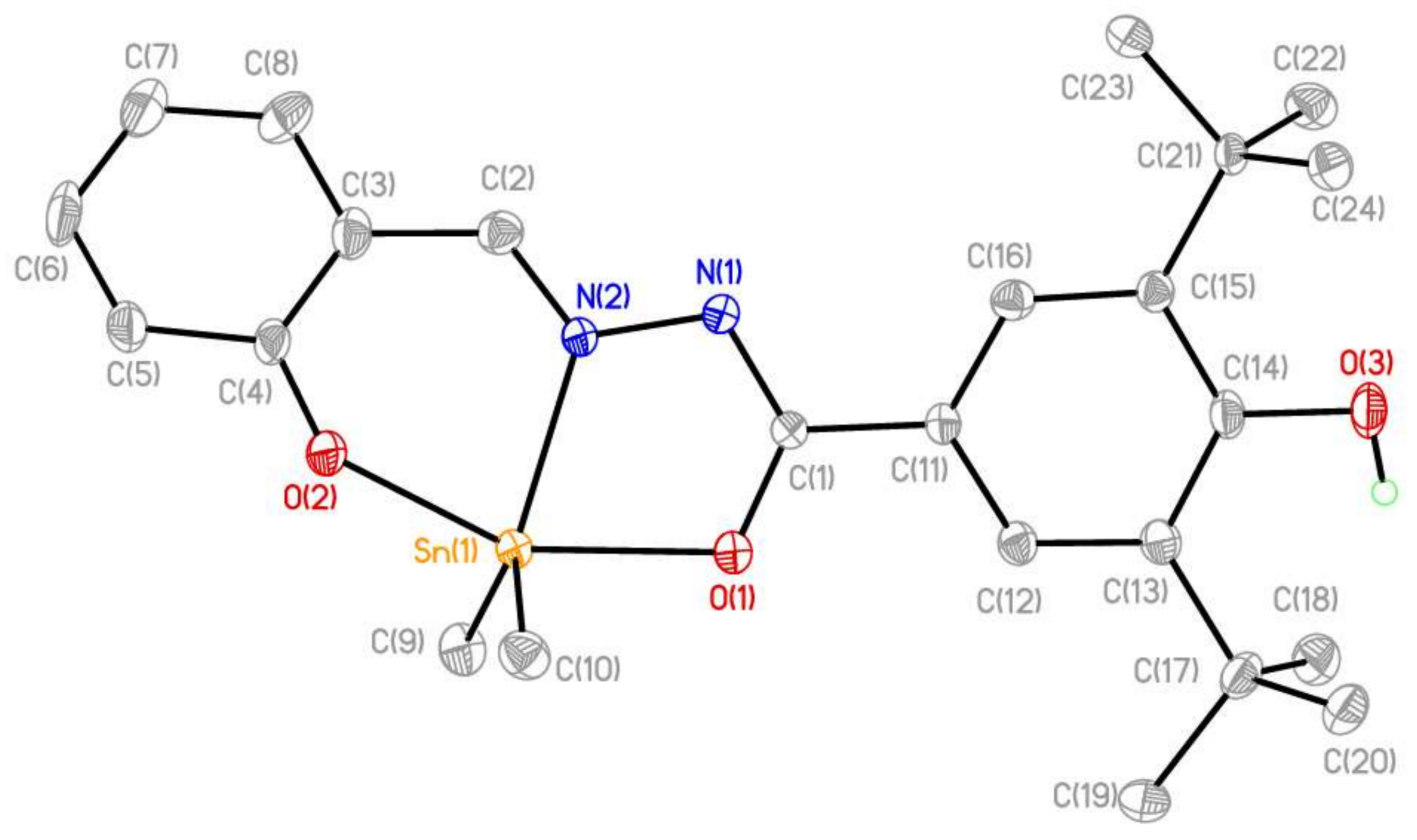
The general view of **2** in representation of atoms by thermal ellipsoids (*p* = 50%).

The structure of complex **2** was determined by X-ray analysis. The tin centre in **2** is five-coordinate within a C_2_NO_2_ donor set derived from *N*,*O*,*O* atoms of the tridentate Schiff ligand and two methyl-C atoms (Figure 1). The coordination polyhedron of tin in complex **2** is significantly distorted. That can be illustrated by the values of τ that are used for the description of five coordination [28]. In **2**, τ is 0.47—almost in the middle between two extreme values—0.0 for an ideal square-pyramid and 1.0 for an ideal trigonal-bipyramid. The widest angle at the Sn(1) is formed by two phenoxide-*O* atoms and is equal to 153.9(1)°.

It should be noted that τ in complex **2** is significantly different from that in the similar compound dimethyltin(IV) [29] in which tin is characterised by square-pyramidal geometry (τ = 0.13). At the same time, similar intermediate coordination polyhedron is observed in the described diphenyl analogue [29]. Thus, we can conclude that the distortion of the tin polyhedron at least for this type of compound cannot be interrelated with the alkyl/aryl substituent volume and is more sensitive to the crystal packing effects.

### 2.2. Studies of Antioxidant Activity

Since organotins are known to induce oxidative stress, thus damaging the cells [11], the introduction of antioxidant pendants can help to attenuate cellular toxic impact [13]. The ligand **1** bears a 2,6-di-*tert*-butylphenol pendant, possessing antioxidant properties, and therefore the obtained series was investigated in vitro for antioxidant activity as well. Radical scavenging properties were studied in an assay with stable radical 2,2-diphenyl-1-picrylhydrazyl (DPPH-test) and in an enzymatic system xanthine-xanthine oxidase (NBT-test). Lipoxygenase inhibitory activity marks the compound as a potent antioxidant, so it was evaluated in a corresponding assay as well.

#### 2.2.1. DPPH-Test

**Figure 2:**
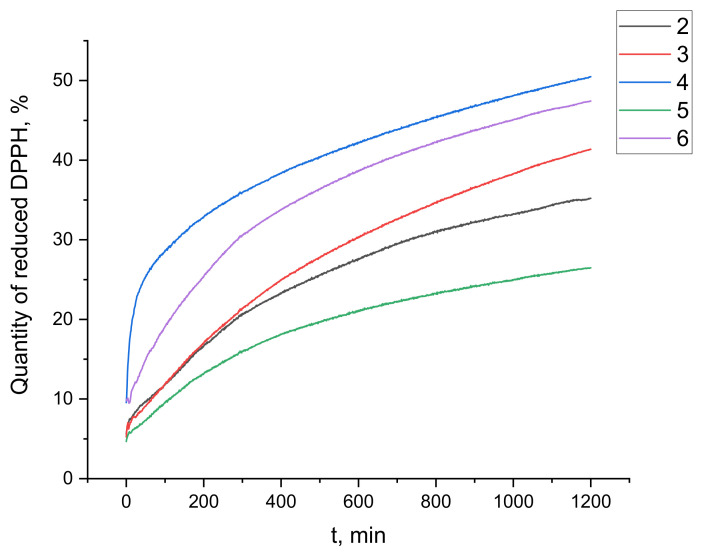
The change of reduced DPPH quantity in a test solution of complexes **2**–**6** during 20 h.

2,2-Diphenyl-1-picrylhydrazyl (DPPH)—is a stable *N*-centered chromophore radical with absorption peak λ_max_ = 517 nm, while its reduced form is pale yellow. Its reduction can be monitored spectrophotometrically and is a well-known [30] and convenient way of evaluating radical scavenging activity. The ligand **1** appeared to be more active than complexes **2**–**6** on its base with EC_50_ = 163 µM. This may be caused by the participation not of only the sterically hindered phenolic group in the process of radical scavenging, but salicylic phenolic group and probably hydrazide fragment as well. The abovementioned structural moieties participate in the coordination of tin atoms, and therefore are excluded from the mechanism of antioxidant action of complexes. Organotin compounds **2**–**6** possess prolonged antioxidant activity which is expressed on long time spans of 20 h (Figure 2). The introduction of tin is known to lower the radical scavenging capacity of corresponding ligands and is estimated as referred previously [31]. The only exception is complex **7**, bearing two 2,6-di-*tert*-butylphenols pendants attached directly to the tin. Since the compound possesses three antioxidant groups in total, its radical activity is superior to its analogues **2**–**6** and even to the ligand **1** itself with EC_50_ = 12.8 µM.

Activity falls in the row as **4** > **6** > **3** > **2** > **5** (Bu > Ph > Et > Me > Bu^t^), thus complexes with aliphatic moieties linked to tin form a pattern, according to which longer alkyl chains seem to improve radical scavenging properties of compounds. This tendency breaks, however, with *tert*-butyltin derivative **5**, demonstrating the lowest antioxidant potency.

#### 2.2.2. NBT-Test

**Scheme 4:**
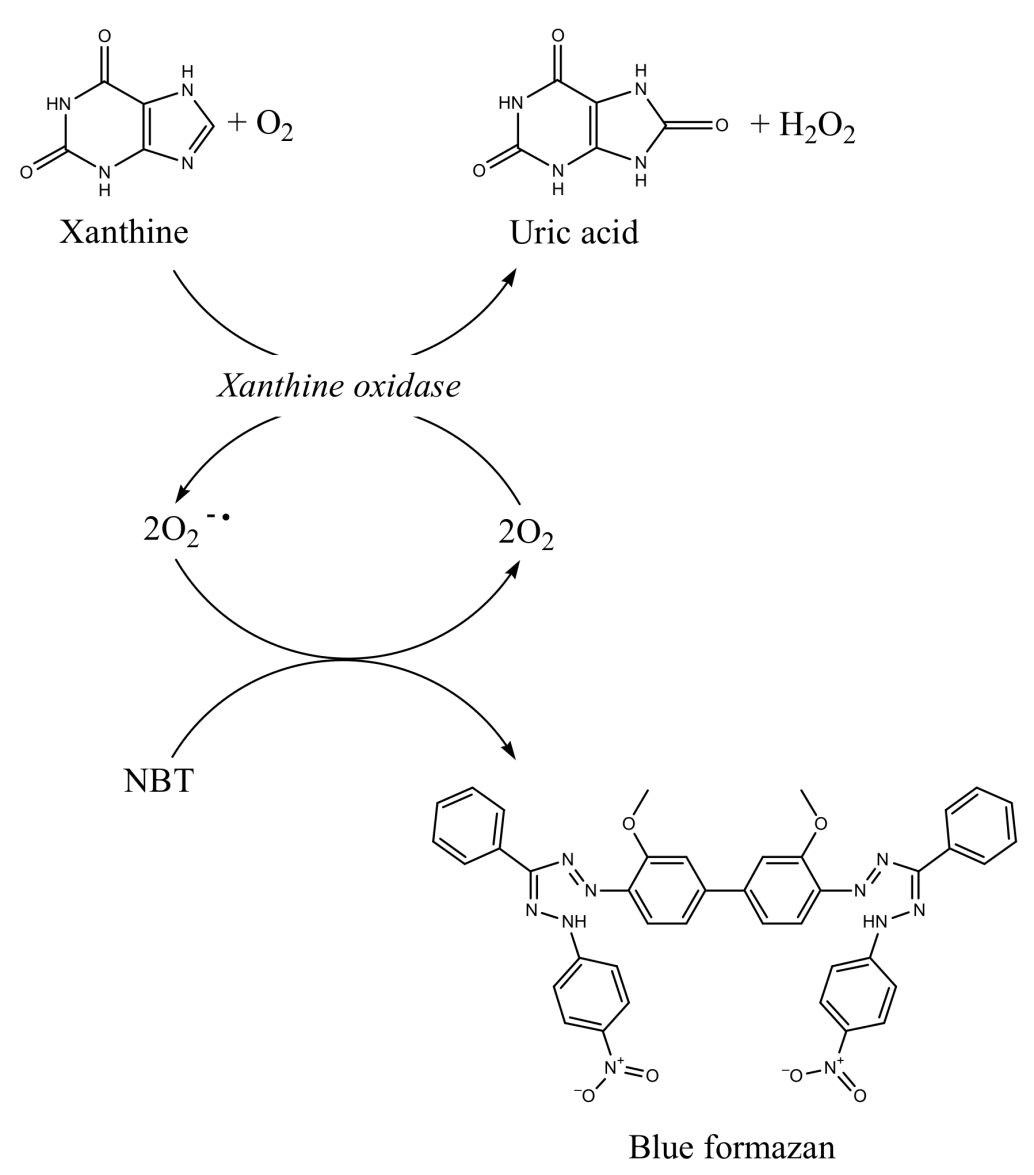
The process of enzymatic O_2_^−•^ generation and its further interaction with NBT.

Xanthine oxidase is molybdenum containing enzyme responsible for purine catabolism in the human organism. It is capable of xanthine oxidation, resulting in uric acid and superoxide radical anion as a side product. The latter one is well-known ROS, and the ability to neutralize it marks the compound as a perspective antioxidant. This is followed by the interaction of O_2_^−•^ and nitro-blue tetrazolium resulting in the cycle opening (Scheme 4), which is accompanied by the color change with λ_max_ = 560 nm and can be monitored spectrophotometrically [32].

Compounds **1**–**5** appeared to be poor O_2_^−•^ scavengers, which may be caused by the reaction kinetics—since most of the compounds demonstrated prolonged antioxidant action, they are not on time to intercept all the radicals. Compounds **6** and **7**, bearing different phenolic moieties attached to tin, show paradoxical results: they stimulate O_2_^−**·**^ formation instead of inhibiting it. Some organotins tend to do this [31] and this may be part of their cytotoxicity mechanism. This is most likely caused by physicochemical reasons rather than enzyme activation.

#### 2.2.3. Lipoxygenase Inhibitory Activity

Lipoxygenases are a group of enzymes catalyzing regioselective oxidation of polyunsaturated fatty acids to corresponding hydroperoxides. Their inhibition is known to suppress the growth of some tumors [33,34,35]; hence, it is a valuable pharmaceutical target [36,37]. Soybean lipoxygenase LOX-1B was used as a model enzyme since it is structurally relative to human 5-lipoxygenases. The influence of synthesized compounds **1**–**7** on the LOX-1B activity was estimated as a formation rate of octadecadienoic acid hydroperoxides (λ_max_ = 234 nm) from the initial acid and was monitored spectrophotometrically (Table 1).

The synthesized compounds showed moderate LOX inhibitory activity with average values of 40–60%. This may be caused by the match of tested molecules and the enzyme’s active site. The inhibition type is to be studied in future research.

#### 2.2.4. Study of Antiglycation Activity

Non-enzymatic glycosylation (glycation) is a pathobiochemical process universal to all living organisms. Glycation is one of the mechanisms of the formation of late complications of diabetes mellitus [38], natural aging, and related diseases [39]. Glycation is available for pharmacological regulation in order to reduce the clinical manifestations of these conditions and their treatment. Different antiglycation agents have different leading mechanisms of action. One of the mechanisms is free radical scavenging which was noted for ligand **1**. An in vitro study provides insight into the potential of compounds to exhibit antiglycation activity and was carried out for ligand **1** and compared to aminoguanidine as a control (Table 2).

According to the results of the study, in terms of IC_50_ value, compound **1** exhibits activity up to 1845 times higher than the reference compound aminoguanidine, while the activity value of ligand **1**, even at maximum concentrations, does not reach the maximum value, which may be due to limited solubility in an aqueous environment.

### 2.3. Studies of Biological Activity

Since we consider the synthesized compounds as potential pharmaceutical agents, we investigated their stability at different pH levels and evaluated their cytotoxicity by a common MTT-test.

#### 2.3.1. Stability Investigation

**Figure 3:**
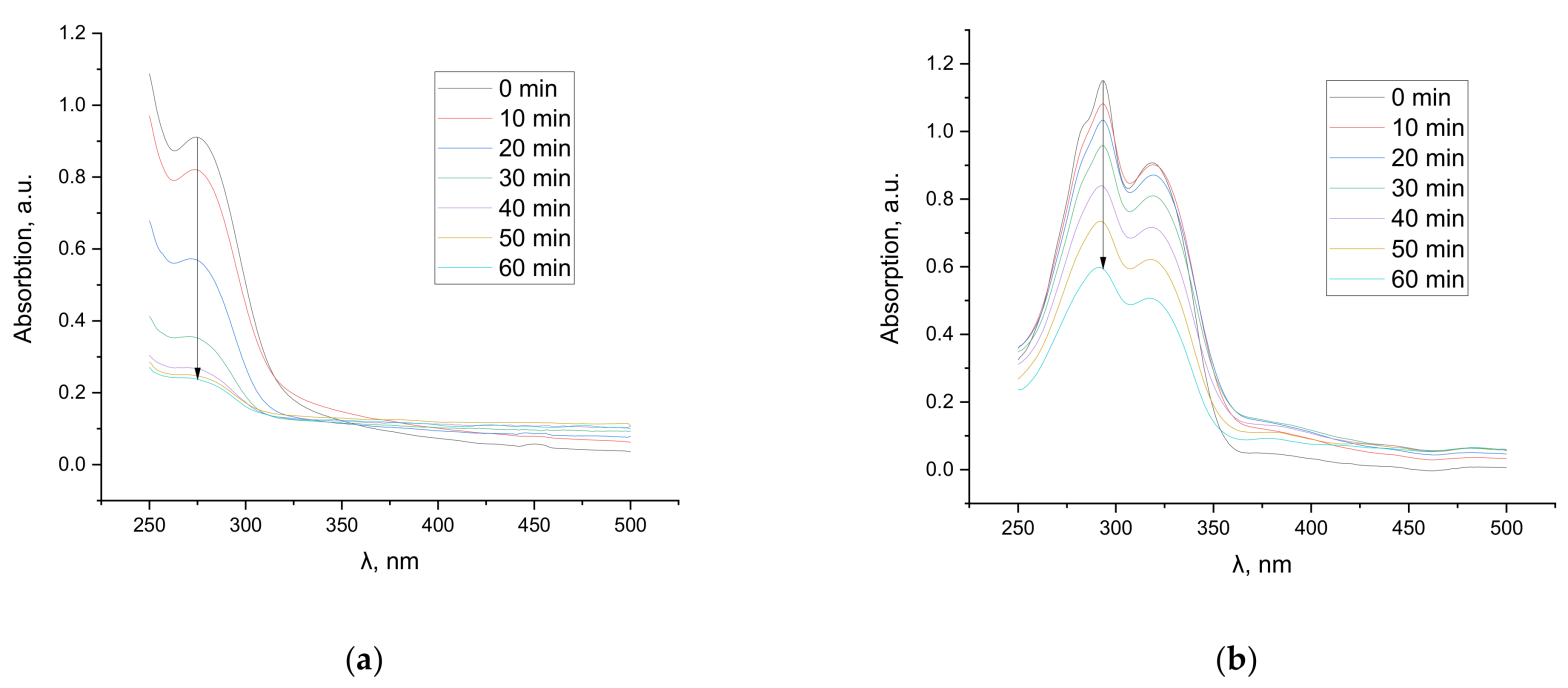
Hydrolysis of ligand **1** for 1 h (**a**) at pH = 5; (**b**) at pH = 7.

**Figure 4:**
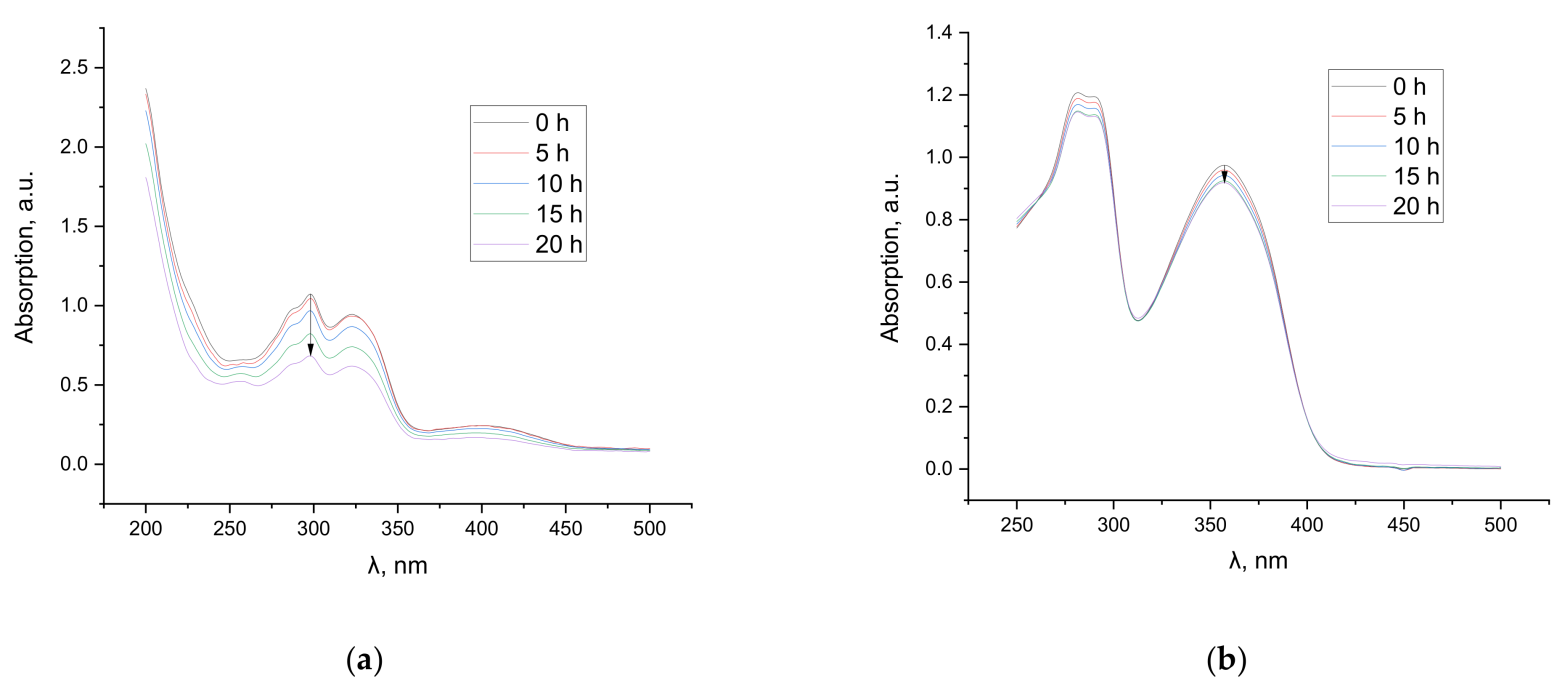
Hydrolysis of complex **3** for 20 h (**a**) at pH = 5; (**b**) at pH = 7.

Investigations of potential drug pharmacokinetics are an important part of the evaluation of its potential efficacy and applicability. On the base level, this can be studied in vitro as a hydrolysis stability assay at different pH levels. Some tumor tissues possess acidic pH, and the organism protects itself by the creation a fibrous sheath. The stability of compounds was estimated as an optical density drop at λ = 250–500 nm at pH = 5 and pH = 7 (Figure 3 and Figure 4).

As it can be seen, ligand **1** decomposes after approximately 1 h of experimentation, probably being hydrolyzed at its azomethine fragment to initial acid hydrazide and salicylic aldehyde. Complexes **2**–**7** on its base, on the other hand, demonstrate vastly more pronounced stability and show similar behavior patterns, showing traces of decomposition only after several hours of an assay. Increased sustainability may be caused by a peculiar polycyclic conjugated system with tin atoms involved in its structure.

#### 2.3.2. MTT-Assay

The organotin toxicity mechanism is a complex and multimodal phenomenon. As was shown in our previous research [8,40], the more probable action routes involve tubulin polymerization inhibition promoted by strong interaction with protein SH groups. This mode of action was confirmed by both experimental studies with isolated tubulin and molecular modeling. Moreover, there is a lot of evidence of oxidative stress induction by organotins and their involvement in mitochondria-induced apoptosis.

Since the investigated compounds are designed as theranostic and cytostatic agents, their toxic impact was evaluated as a standard MTT-test [41]. Cytotoxicity was studied on four cell lines, i.e.,—HCT116 (human colorectal carcinoma), MCF-7 (human breast cancer), A549 (human lung adenocarcinoma), and WI-38 (human fetal lung fibroblast). Obtained results appear to be unorthodox: as it is known, the introduction of an organotin moiety tends to noticeably increase compounds’ toxicity [18,31] and more lipophilic ones do it better [42], but in the presented data pattern turns vice versa (Table 3).

In the experiments carried out, ligand **1** and its diethyltin derivative **3** demonstrate the lowest IC_50_ values with dimethyltin compound **2** falling short of them. Then comes the dibutyltin compound **4**, which still shows good results on the MCF-7 cell line. The activity of **6** and **7** is less pronounced, while di-*tert*-butyltin complex **5** possesses the highest IC_50_ values. It is known, that there is an optimum of organotins lipophilicity, after which their toxicity starts to decrease [43]. This principle is obviously manifested in the presented data.

According to the comprehensive review made by one of the leading scientific groups in the field of organometallic chemistry [44], the average toxicity for organotins on the MCF-7 cell line is 42.46 ± 30.25 μM for complexes possessing *O*-donor and 4.86 ± 2.10 μM for the ones possessing *N*-donor ligands. Synthesized compounds possess combined *O*,*N*,*O* coordination types and it can be concluded that involving *N*-atoms in the coordination group raises the cytotoxic properties of the resulting agent. Our scientific group has carried out research on dialkyltins with antioxidant and bile acid ligands [45]. The complexes reported in the present article in general demonstrate comparable activity, but have slightly more pronounced selectivity to some cancer cell lines. Additionally, dibutyltin derivatives with antioxidant moiety from the article mentioned above [45] and compound **4** from this study demonstrate similar selectivity for the MCF-7 cell lines.

**Figure 5:**
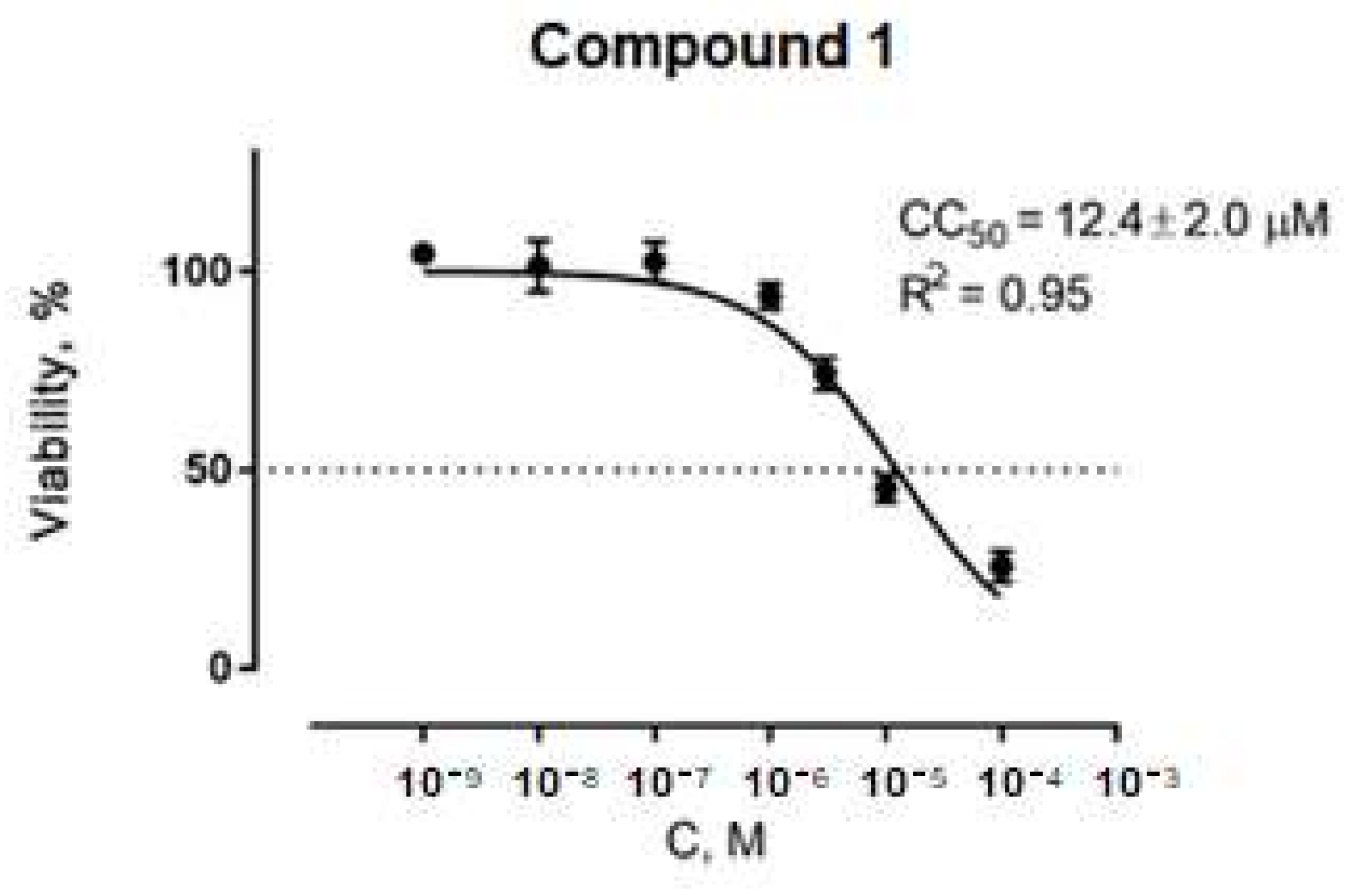
An effect of compound **1** at concentrations of 0.001–100 µM on the metabolic activity (viability) of HepG2 cells during 48 h incubation in the MTT test.

Cytotoxic properties of **1** are unusual, so an additional cytotoxicity assay with HepG2 cells was carried out and CC_50_ values were estimated (Figure 5).

For compound **1** in the concentration range of 3–300 μM, the ability to significantly reduce the metabolic activity which correlates with the viability of HepG2 cells during 48-h incubation in the MTT test is noted. The minimal concentration that significantly suppresses vital activity by 25.8 ± 4.0% relative to the control value was 3 µM. At the same time, the CC_50_ value for compound **1** was 12.4 ± 2.0 µM. The fact that ligand **1** itself has cytotoxic properties can be interpreted as the existence of a pharmacological target, specific to **1**. The detection of potential targets for ligand **1** involves research on cell cycle influence and many other assays, and will be carried out in future studies.

### 2.4. Luminescence Studies

The obtained compounds demonstrated luminescent properties both as solid powders and as solutions. This, in combination with their cytotoxic properties, makes them prospective candidates for theranostic agent materials, which can combine cancer therapy and diagnostic properties, i.e., luminescent bioimaging. Although organotin complexes possessing similar *O*,*N*,*O* coordination types have been reported before [27,29] no investigations of their luminescent properties were carried out. It is hard to say if it is related to structural peculiarities or other factors.

**Figure 6:**
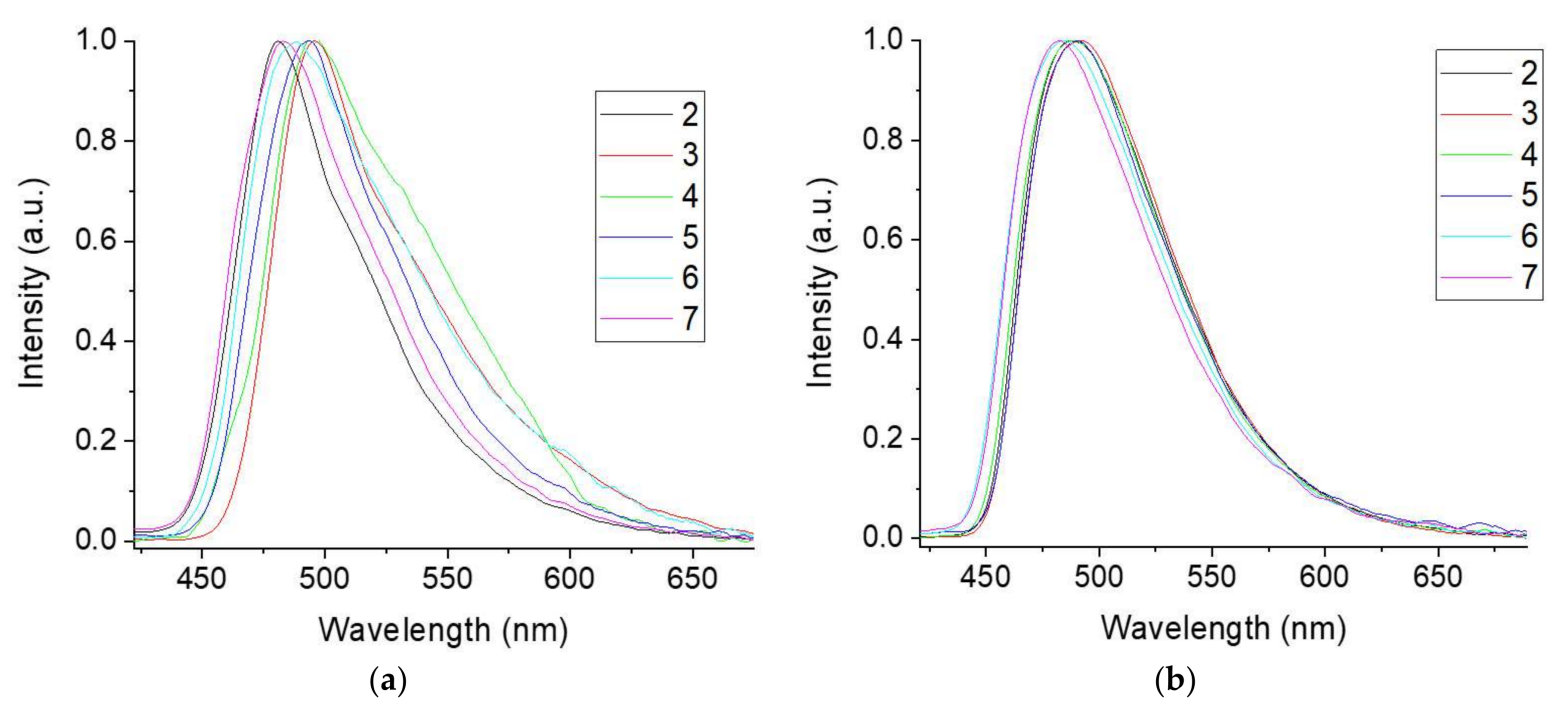
Luminescence spectra of the samples **2**–**7**: (**a**) powders (sample **4** was an oil), (**b**) DMSO solutions (1000 µM).

The obtained solid organotin complexes demonstrated intense luminescence in the visible range, centered in the range of 481–495 nm (Figure 6). Their quantum yields reached 20%, which together with their high absorption (Table 4) makes them highly emissive even in powders.

The obtained quantum yields correlate with the nature of the substituent in the obtained organotin complexes. So, the lowest values of 4–8% were reached for the compounds with bulky substituents R = Bu, Bu^t^, and Ph, while for the smaller R = Me, Et higher values of 14% were reached. The highest value of 20%, though, was also obtained for compound **7** with an aromatic substituent; this can relate to the additional contacts due to the presence of the OH-groups. Despite this matter being deserving of further study, such a correlation speaks in favor of the quantum yield dependence on the intermolecular interactions.

Indeed, the quantum yield values align when moving from powders to solutions, where intermolecular interactions do not play such a role. The quantum yields of the 1000 μM DMSO solutions vary from 26% to 39%, and the highest value of 39% is now reached for compound **2**. It is also clear that the quantum yield values increase after the dissolution, which is typical for organic compounds subject to concentration quenching. The latter phenomenon is common for the organic emitter. A high concentration of the emitter causes effective energy transfer between activator molecules, quenching the photon emission. The decrease of the concentration up to a certain value decreases concentration quenching efficiency, thus increasing the quantum yield.

The quantum yield dependence on concentration was studied to confirm the concentration quenching role. It revealed that the concentration decrease down to 100 μM results in a further increase of the quantum yields. The values of 37–67% were reached, and the highest value of 67% was now obtained for compound **4** with R = Bu, which demonstrated the lowest quantum yield before dissolution. Further concentration decline down to 10 μM resulted in the decrease of the quantum yields due to the dilution (Table 4).

Thus, the studied compounds demonstrated intense luminescence in both powders and DMSO solution, including the range of 10 μM, in which some of these compounds should be used according to the cytotoxicity studies.

## 3. Materials and Methods

Sigma-Aldrich (Merck KGaA, Darmstadt, Germany) and ABCR (ABCR GmbH, Karlsruhe, Germany) provided 2-Hydroxybenzaldehyde (≥98%), triethylamine (≥99%), Me_2_SnCl_2_ (97%), Et_2_SnCl_2_ (97%), Bu_2_SnCl_2_ (96%), Bu^t^_2_SnCl_2_ (98%), and Ph_2_SnCl_2_ (96%), which were used with no further purification. The 3,5-di-*tert*-butyl-4-hydroxybenzoic hydrazide was prepared according to the known method [26]. The synthesis of bis-(2,6-di-*tert*-butylphenol)tin(IV) dichloride was carried out according to the described method [13]. The solvents (EtOH (95%), MeOH, Et_2_O, toluene, and petroleum ether (b.p. 40–70 °C)) were used as supplied.

NMR spectra were measured on a Bruker AMX-400 spectrometer in DMSO-*d*_6_ (^1^H, 400 MHz; ^13^C, 100.6 MHz; ^119^Sn, 149.15 MHz). IR spectra of dry compounds were recorded on ThermoNicolet IR200 (Thermo Fisher Scientific, Waltham, MA, USA).

### 3.1. General Synthesis of Ligand (L)

The 2-Hydroxybenzaldehyde (0.2 mmol) was added to a solution of 3,5-di-*tert*-butyl-4-hydroxybenzohydrazide (0.2 mmol) in 5 mL of EtOH followed by stirring and heating for 24 h. The solvent was removed in vacuo, and the residue was washed with diethyl ether (3 × 5 mL) and H_2_O (3 × 5 mL), dried in the air, recrystallized from Et_2_O, and isolated as a colorless powder.

#### Di-*tert*-butyl-4-hydroxy-*N*′-(2-hydroxybenzylidene)-benzohydrazine (**1**)

Yield 61%. Mp 265–269 °C.

IR, cm^−1^: 3615.4–3604.8 (ν OH, bound); 3222.5 (ν NH); 3068.2–2912.0 (ν CH); 1644.0 (ν C=O); 1622.8, 1609.3 (ν C-C, Ar); 1549.0 (ν C=N); 1489.3; 1435.7; 1359.6; 1305.1; 1273.3; 1236.6.

^1^H NMR (DMSO-*d_6_*, δ, ppm): 1.42 (s, 18H, 2 Bu^t^); 6.91 (m, 2H, 2 CH-Ar-SA); 7.28 (t, 1H, CH-Ar-SA, ^3^*J*_HH_ = 14.9 Hz); 7.51 (dd, 1H, CH-Ar-SA, ^3^*J*_H-H_ = 8.4, 1.0 Hz); 7.60 (s, 1H, OH); 7.67 (s, 2H, H-Ar); 8.62 (s, 1H, CH); 11.42 (s, 1H, HO-Ar-SA); 11.87 (s, 1H, NH).

^13^C NMR (DMSO-*d_6_*, δ, ppm): 30.16 (C(CH_3_)_3_); 34.67 (C(CH_3_)_3_); 116.43, 118.78, 119.33, 129.59 (C_2′_-C_5′_-Ar-SA); 123.82 (C_1_-Ar); 124.61 (C_2_-Ar); 131.18 (C_6′_-Ar-SA); 134.74 (C_1′_-Ar-SA); 138.46 (C_3_-Ar); 147.71 (C_4_-Ar); 157.50 (C(O)NH); 163.55 (CH(N)).

Elemental analysis, for C_22_H_28_N_2_O_3_ calcld (%): C, 71.70; H, 7.67; N, 7.60. Found (%): C, 71.58; H, 7.56; N, 7.56.

### 3.2. General Synthesis of Complexes (***2***–***7***)

Complexes **2**–**7** were synthesized according to the general method. To the mixture of ligand 1 (2 mmol) and triethylamine (2 mmol) in toluene corresponding organotin chloride (1 mmol) was added. The reaction mixture was stirred for 1 h at rt. Then the solvent was removed in vacuo, the residue was washed with diethyl ether (3 × 3 mL), Et_2_O was removed in vacuo. The products were isolated as vivid yellow-green powders except for complex **4**, which was isolated as yellow-green oil.

#### 3.2.1. Me_2_SnL (**2**)

Yield 78%. Mp 211–215 °C.

IR, cm^−1^: 3630.8 (ν OH, free); 2959.2–2870.0 (ν CH); 1607.9 (ν C=N); 1544.2 (ν C-C, Ar); 1500.4; 1468.5; 1380.3; 1356.7.

^1^H NMR (DMSO-*d*_6_, δ, ppm): 0.67 (s, 6H, 2 CH_3_, ^2^*J*_Sn-H_ = 87 Hz); 1.39 (s, 18H, 2 Bu^t^); 6.65 (m, 2H, 2 CH-Ar-SA); 7.25 (t, 1H, CH-Ar-SA, ^3^*J*_H-H_ = 15 Hz); 7.39 (d, 1H, CH-Ar-SA, ^3^*J*_H-H_ = 8 Hz); 7.41 (s, 1H, OH); 7.83 (s, 2H, 2 CH-Ar); 8.81 (s, 1H, CH(N), ^2^*J*_Sn-H_ = 37 Hz).

^13^C NMR (DMSO-*d*_6_, δ, ppm): 4.82 (2 CH_3_); 30.23 (C(CH_3_)_3_); 34.55 (C(CH_3_)_3_); 79.25 (C_6′_-Ar-SA); 116.28, 117.42, 120.73, 120.99 (C_2′_-C_5′_-Ar-SA); 124.13 (C_2_-Ar); 124.26 (C_1_-Ar); 134.46 (C(O)N); 134.92 (C_1′_-Ar-SA); 138.23 (C_3_-Ar); 139.78 (C_4_-Ar); 165.67 (CH(N)).

^119^Sn (DMSO-*d*_6_, δ, ppm): −206.59.

Elemental analysis, for C_24_H_32_N_2_O_3_Sn calcld (%): C, 55.94; H, 6.27; N, 5.44. Found (%): C, 56.02; H, 6.26; N, 5.34.

#### 3.2.2. Et_2_SnL (**3**)

Yield 88%. Mp 145–147 °C.

IR, cm^−1^: 3619.3 (ν OH, free); 2953.0–2871.0 (ν CH); 1604.5 (ν C=N); 1540.9, 1504.7 (ν C-C, Ar); 1470.0; 1380.3; 1354.8.

^1^H NMR (DMSO-*d*_6_, δ, ppm): 1.08 (t, 6H, 2 CH_3_, ^3^*J*_H-H_ = 16 Hz); 1.34 (br. Q, 4H, 2 CH_2_, ^3^*J*_H-H_ = 21.0 Hz); 1.39 (s, 18H, 2 Bu^t^); 6.62 (m, 2H, CH-Ar-SA); 7.22 (td, 1H, CH-Ar-SA, ^3^*J*_H-H_ = 17.2, 1.8 Hz); 7.36 (dd, 1H, CH-Ar-SA, ^3^*J*_H-H_ = 9.4, 1.7 Hz); 7.38 (s, 1H, OH); 7.85 (s, 2H, 2 CH-Ar); 8.82 (s, 1H, CH(N), ^2^*J*_Sn-H_ = 30.1 Hz).

^13^C NMR (DMSO-*d*_6_, δ, ppm): 9.84 (2 CH_3_); 18.71 (2 CH_2_); 30.65 (C(CH_3_)_3_); 34.95 (C(CH_3_)_3_); 79.25 (C_6′_-Ar-SA); 116.28, 117.42, 120.73, 120.99 (C_2′_-C_5′_-Ar-SA); 124.13 (C_2_-Ar); 124.26 (C_1_-Ar); 134.46 (C(O)N); 134.92 (C_1′_-Ar-SA); 138.62 (C_3_-Ar); 139.78 (C_4_-Ar); 165.67 (CH(N)).

^119^Sn (DMSO-*d*_6_, δ, ppm): −255.65.

Elemental analysis, for C_26_H_36_N_2_O_3_Sn calcld (%): C, 57.47; H, 6.69; N, 5.16. Found (%): C, 57.34; H, 6.84; N, 5.14.

#### 3.2.3. Bu_2_SnL (**4**)

Yield 74%. Oil.

^1^H NMR (DMSO-*d*_6_, δ, ppm): 0.74 (m 6H, 2 CH_3_); 1.24 (m, 4H, 2 CH_2_); 1.38 (s, 18H, 2 Bu^t^); 1.49 (m, 8H, 4 CH_2_); 6.62 (m, 2H, CH-Ar-SA); 7.23 (td, 1H, CH-Ar-SA, ^3^*J*_H-H_ = 17.4, 1.7 Hz); 7.36 (dd, 1H, CH-Ar-SA, ^3^*J*_H-H_ = 10.0, 1.7, Hz); 7.38 (s, 1H, OH); 7.83 (s, 2H, 2 CH-Ar); 8.85 (s, 1H, CH(N), ^2^*J*_Sn-H_ = 37.5 Hz).

^13^C NMR (DMSO-*d*_6_, δ, ppm): 18.72, 29.86, 30.88, 39.69 (Bu); 31.85 (C(CH_3_)_3_); 35.38 (C(CH_3_)_3_); 121.29, 122.44, 126.00 (C_2′_-C_4′_-Ar-SA); 129.21, 129.49 (C_1_-C_2_-Ar) 139.49 (C(O)N); 139.61 (C_1′_-Ar); 143.40 (C_3_-Ar); 161.93 (C_5′_-Ar-SA); 164.99 (C_4_-Ar); 171.61 (C_6′_-Ar-SA); 174.20 (CH(N)).

^119^Sn (DMSO-*d*_6_, δ, ppm): −225.09.

Elemental analysis, for C_30_H_44_N_2_O_3_Sn calcld (%): C, 60.11; H, 7.41; N, 4.67. Found (%): C, 59.99; H, 7.49; N, 4.80.

#### 3.2.4. Bu^t^_2_SnL (**5**)

Yield 66%. Mp 186–190 °C.

IR, cm^−1^: 3629.4 (ν OH, free); 2957.8–2849.3 (ν CH); 1611.7 (ν C=N); 1545.2, 1505.2 (ν C-C, Ar); 1469.5; 1377.9; 1355.2.

^1^H NMR (DMSO-*d*_6_, δ, ppm): 1.24 (s, 18H, 2Bu^t^); 1.38 (s, 18H, 2Bu^t^); 6.73 (m, 2H, CH-Ar-SA); 7.28 (td, 1H, CH-Ar-SA, ^3^*J*_H-H_ = 16.8, 1.5 Hz); 7.42 (dd, 1H, CH-Ar-SA, ^3^*J*_H-H_ = 9.4, 1.5 Hz); 7.43 (s, 1H, OH); 7.87 (s, 2H, 2 CH-Ar); 8.97 (s, 1H, CH(N), ^2^*J*_Sn-H_ = 41.1 Hz).

^13^C NMR (DMSO-*d*_6_, δ, ppm): 29.70 (Sn-C(CH_3_)_3_); 30.56 (C(CH_3_)_3_); 34.90 (C(CH_3_)_3_); 39.84 (C(CH_3_)_3_); 116.99, 117.52, 121.33 (C_2′_-C_4′_-Ar-SA); 124.28, 124.32 (C_1_-C_2_-Ar) 134.91 (C(O)N); 135.08 (C_1′_-Ar); 138.73 (C_3_-Ar); 157.38 (C_5′_-Ar-SA); 160.96 (C_4_-Ar); 167.50 (C_6′_-Ar-SA); 169.39 (CH(N)).

^119^Sn (DMSO-*d*_6_, δ, ppm): −282.59.

Elemental analysis, for C_30_H_44_N_2_O_3_Sn calcld (%): C, 60.11; H, 7.41; N, 4.67. Found (%): C, 60.08; H, 7.39; N, 4.75.

#### 3.2.5. Ph_2_SnL (**6**)

Yield 89%. Mp 139–143 °C.

IR, cm^−1^: 3620.2 (ν OH, free); 3050.8–2870.5 (ν CH); 1606.9 (ν C=N); 1543.7, 1500.4 (ν C-C, Ar); 1470.4; 1380.8; 1355.7.

^1^H NMR (DMSO-*d*_6_, δ, ppm): 1.41 (s, 18H, 2 Bu^t^); 6.70 (td, 1H, CH-Ar-SA, ^3^*J*_H-H_ = 15.5, 1.2 Hz); 6.89 (d, 1H, CH-Ar-SA, ^3^*J*_H-H_ = 7.5 Hz); 7.32 (m, 13H, 2Ph, 2CH-Ar-SA, OH); 7.95 (s, 2H, 2 CH-Ar); 8.77 (s, 1H, CH, CH(N), ^2^*J*_Sn-H_ = 52.5 Hz).

^13^C NMR (DMSO-*d*_6_, δ, ppm): 30.62 (C(CH_3_)_3_); 34.97 (C(CH_3_)_3_); 117.29, 118.33, 121.95, 124.49, 124.59, 125.75, 128.64, 128.83, 129.33, 129.49, 134.92, 135.33 (2 Ph); 137.77 (C(O)N); 138.79 (C_1′_-Ar); 146.16 (C_3_-Ar); 157.46 (C_5′_-Ar-SA); 157.77 (C_4_-Ar); 166.28 (C_6′_-Ar-SA); 168.57 (CH(N)).

^119^Sn (DMSO-*d*_6_, δ, ppm): −404.94.

Elemental analysis, for C_34_H_36_N_2_O_3_Sn calcld (%): C, 63.86; H, 5.69; N, 4.38. Found (%): C, 63.92; H, 5.66; N, 4.26.

#### 3.2.6. (2,6-Di-*tert*-butylphenol)_2_SnL (**7**)

Yield 69%. Mp > 310 °C.

IR, cm^−1^: 3627.0 (ν OH, free); 2955.4–2870.5 (ν CH); 1605.9 (ν C=N); 1543.7, 1504.7 (ν C-C, Ar); 1469.5; 1427.6; 1377.9; 1353.8; 1235.7; 1147.4; 1121.9.

^1^H NMR (DMSO-*d*_6_, δ, ppm): 1.30 (s, 36H, 2 Bu^t^); 1.42 (s, 18H, 2 Bu^t^); 6.77 (t, 1H, CH-Ar-SA, ^3^*J*_H-H_ = 15.8 Hz); 6.95 (d, 1H, CH-Ar-SA, ^3^*J*_H-H_ = 8.1 Hz); 7.13–7.26 (m, 4H, CH-Ar, CH-Ar-SA); 7.40 (dt, 1H, CH-Ar-SA, ^3^*J*_H-H_ = 17.2, 1.5 Hz); 7.49 (dd, 1H, CH-Ar-SA, ^3^*J*_H-H_ = 9.7, 1.7 Hz); 7.52 (s, 1H, OH); 7.55 (s, 4H, CH-Ar, ^2^*J*_Sn-H_ = 82.2 Hz); 8.02 (s, 2H, OH); 8.92 (s, 1H, CH(N), ^2^*J*_Sn-H_ = 52.6 Hz).

^13^C NMR (DMSO-*d*_6_, δ, ppm): 35.28 (C(CH_3_)_3_); 35.56 (C(CH_3_)_3_); 39.61 (C(CH_3_)_3_); 39.73 (C(CH_3_)_3_); 122.30, 125.25, 128.87, 129.18, 129.56, 130.50, 133.38, 134.08, 135.70, 136.90, 139.91, 140.77, 142.52, 143.58, 144.31, 150.76, 151.92, 154.03, 161.14, 162.29.

^119^Sn (DMSO-*d*_6_, δ, ppm): −336.16.

Elemental analysis, for C_54_H_78_N_2_O_5_Sn calcld (%): C, 67.98; H, 8.26; N, 2.94. Found (%): C, 67.94; H, 8.29; N, 2.91.

### 3.3. Crystallographic Data Collection and Structure Determination

Yellow crystals of **2** (C_24_H_32_N_2_O_3_Sn, M = 515.20) are monoclinic, space group P2_1_/n, at 104(2) K: a = 6.0929(4), b = 11.4521(10), c = 32.930(3) Å, β = 90.909(3)°, V = 2297.4(3) Å^3^, Z = 4 (Z′ = 1), d_calc_ = 1.490 g**^.^**cm^−3^, μ(MoKα) = 11.39 mm^−1^, F(000) = 1056. Intensities of 21,613 reflections were collected at 104(2) K on a Bruker Quest D8 diffractometer equipped with a Photon-III area-detector (shutterless ϕ- and ω-scan technique), using Mo K_a_-radiation. 6123 independent reflections [R_int_ = 0.0837] were used in further refinement. Considering the highly anisotropic shape of the crystals, the absorption correction was performed using a multiscan routine as implemented in SADABS (Version 2016/2) [46]. The structure was solved by direct methods using SHELXT [47] and refined on *F*^2^ using SHELXL-2018 [48]. Positions of all atoms were found from the electron density-difference map. Atoms were refined with individual anisotropic (non-hydrogen atoms) or isotropic (hydrogen atoms) displacement parameters. The OH group is disordered by two and three positions. The refinement converged to wR2 = 0.1260 and GOF = 1.031 for all independent reflections (R1 = 0.0546 was calculated against F for 4790 observed reflections with I > 2σ(I)). The SHELXTL program suite (XSHELL version, George M. Sheldrick, Goettingen, Germany) was used for molecular graphics. Atomic coordinates, bond lengths and angles, and thermal parameters have been deposited at the Cambridge Crystallographic Data Center with deposition number CCDC 2215030.

### 3.4. Antioxidant Assay

#### 3.4.1. DPPH Assay

The activity of synthesized compounds as radical scavengers was estimated spectrophotometrically at λ_max_ = 517 nm using stable radical 2,2-diphenyl-1-picrylhydrazyl (Sigma-Aldrich) according to the known procedure [49,50]. The reaction mixture contained DPPH (0.75 mL, 0.2 mM) and a solution of the test compound in EtOH (0.75 mL, 0.2 mM), and was put in 1 cm glass cuvettes. The measurements were carried out for 20 h and the results were calculated using Microsoft Excel 2010. Antioxidant activity was expressed as the percentage of reduced DPPH according to the Equation (1):

I (%) = ((A_0_ − A_1_)/A_0_) × 100 (1)

where A_0_ is the absorbance of the control 0.1 mM DPPH in EtOH, ε DPPH = 1.15·× 10^4^, and A_1_ is the absorbance of the reaction mixture in the presence of the test compound.

#### 3.4.2. Enzymatic Generation of the Superoxide Radical Anion О_2_^−•^ in the Xanthine—Xanthine Oxidase System (NBT Assay)

The influence of compounds on superoxide radical anion О_2_^−•^ generated in the enzymatic system xanthine-xanthine oxidase was estimated as the amount of nitro-blue tetrazolium (NBT) reduced to formazan [32]. The assay was carried out in 96-well plates. The total volume of a single well was 0.3 mL and consisted of 0.27 mL of carbonate buffer (40 mM, pH 10.0) containing EDTA (0.1 mM), 0.006 mL of xanthine (10 mM) in carbonate buffer, 0.003 mL of 0.5% bovine serum albumin in water, 0.003 mL of NBT chloride (2.5 mM) in water, and 0.006 mL of a solution of the studied compound in DMSO (5 mM). Xanthine oxidase (0.012 mL, 0.004 units) in buffer was added to the mixture at rt to start the reaction and the absorption at λ_max_ = 560 nm was recorded for 600 s. The control experiment was performed in the presence of 0.006 mL DMSO without a compound. All experiments were performed in triplicate.

Inhibition was expressed by the Equation (2):

I (%) = (ν_0_/ν_0_′) × 100%, (2)

where v_0_ and v_0_′ are initial rates of the enzymatic reaction in the presence and absence (control) of the compounds under study, respectively.

The initial rate (v_0_ and v_0_′) was calculated by the Equation (3):

ν_0_ = ΔС/Δt = ΔA/(Δt × ε) = tgα/(Δt × ε), (3)

A_1_ is the absorbance in the presence of the testing compound at the end of the reaction (600 s), and A_0_ is the absorbance of the blank solution. ΔA is the difference between A_1_ and A_0_. All experiments were performed in triplicate.

#### 3.4.3. Inhibition of Lipoxygenase (LOX 1-B)

The lipoxygenase activity was evaluated spectrophotometrically. The concentrations of linoleic acid oxidation products, isomeric hydroperoxides, were measured at λ_max_ = 234 nm (ε = 25,000 L mol^−1^ cm^−1^) with a 96-well microplate spectrophotometer Multiskan Go (Thermo Fisher Scientific, Waltham, MA, USA). The analyzed solution contained 30 µL borate buffer (pH 9.0), 100 µL linoleic acid (0.45 mM) in borate buffer, and 3 µL 1 mM solution of the test compound in DMSO. The reaction was initiated by the addition of 17 µL of lipoxygenase (500 U) solution in borate buffer. The measurements were performed for 5 min at 20 °C.

The inhibition rate I (%) of lipoxygenase was determined by the Equations (2) and (3), where A_0_ is the absorbance of the control solution, and A_1_ is the absorbance of the reaction mixture in the presence of the tested compound 5 min after the beginning of the reaction. ΔA is the difference between A_1_ and A_0_. All experiments were performed in triplicate.

#### 3.4.4. Study of Antiglycation Activity

The glycation reaction was carried out in a phosphate buffer solution of 0.05 M, pH 7.4. Composition of the reaction medium: 0.36 M glucose solution and 1 mg/mL BSA (fraction V). The test compounds were dissolved in 99% DMSO (final concentration in the reaction medium ~3%). The activity of compound **1** was studied in the concentration range of 1000–0.1 μM, and the activity of aminoguanidine was determined at concentrations of 10,000–1000 μM. Control samples contained an equivalent volume of solvent. The samples were incubated for 24 h at 60 °C.

Data registration was carried out by the spectrofluorimetric method, determining AGE by specific fluorescence at excitation/emission wavelengths of 370/440 nm (spectrofluorimeter M 200 PRO, TECAN Austria GmbH, Grödig, Austria).

In order to exclude false positive results for compounds suppressing AGE fluorescence due to interference, the obtained data were logarithmically normalized according to Equation (4):

Fly(log) = 10^(log10(Exp)−log10(Blank))^ − 1 (4)

where Fly(log) is the normalized AGE fluorescence intensity, log10(Exp) and log10(blank) are the decimal logarithms of the actual fluorescence levels of glycated and corresponding non-glycated samples (both containing the test compound and controls).

The activity of other compounds (both non-fluorescent and fluorescent at the wavelengths used) was expressed by the Equation (5):

Fly(lin) = Exp − Blank(5)

where Fly(lin) is the fluorescence intensity of AGEs, Exp and blank are the actual fluorescence levels of glycated and corresponding non-glycated samples (both those containing the test compound and controls).

Determination of activity expressed as % suppression of fluorescence of AGE, was made according to the Equation (6):

% = 100 − (Fly(Exp) × 100/Fly(Contr))(6)

where Fly(Exp) and Fly(Contr) are the CPG fluorescence intensity of experimental and control samples, respectively (log-normalized or non-log-normalized).

Mathematical data processing was carried out using Microsoft Excel software (Microsoft, Redmond, WA, USA).

### 3.5. Biological Studies

#### 3.5.1. Stability Studies

The stability of synthesized compounds was estimated spectrophotometrically. To 1.5 mL of either phosphate buffer with pH = 5.0 or phosphate buffer with pH = 7.4, 300 µL of 2 mM test solution in MeOH was added. Then adsorption spectra at λ = 250–500 nm were recorded for 20 h. Graphs were built using OriginPro 2019b.

#### 3.5.2. MTT Assay

HCT-116 (colon carcinoma), MCF-7 (breast adenocarcinoma), A-549 (lung adenocarcinoma) cell lines, and WI-38 (cell line composed of fibroblasts) were derived from The European Collection of Authenticated Cell Cultures (ECACC, Salisbury, UK), cultured in DMEM medium (Gibco™, Dublin, Ireland) supplemented with 10% fetal bovine serum (Gibco™, Brasilia, Brazil). The cells were cultured in an incubator at 37 °C in a humidified 5% CO_2_ atmosphere and subcultured two times a week.

The toxicity of compounds was studied on cultured cell lines according to standard MTT protocol [41,51].

### 3.6. Luminescence Properties

The registration of luminescence spectra of powders and solutions in the visible range as well as the measurement of quantum yields were carried out with Horiba FluoroMax Plus, with xenon lamp as excitation source at room temperature; excitation was performed through a ligand (λ_ex_ = 365 nm). The quantum yield was measured by the absolute method in the integrating sphere.

## 4. Conclusions

A series of novel organotin complexes with polydentate chelating antioxidant ligands was synthesized and characterized by physicochemical analysis methods. Their radical scavenging activity was estimated by the DPPH test. It was shown that the antioxidant properties of the ligand are in general higher than that of complexes, inferior only to its derivative with additional 2,6-di-*tert-*butylphenol pendants linked to tin. This demonstrates, that coordination groups of the ligand participate in the mechanism of radical scavenging as well. Nonetheless, synthesized compounds show almost zero activity on the NBT-test, which may be caused by the reaction kinetics. Additionally, complexes possessing phenyl moieties linked to tin tend to boost the O_2_^−^ formation, which is probably caused by the physicochemical peculiarities of the process. For the ligand alone, the pronounced ability to suppress AGE formation was demonstrated, which correlates with its radical scavenging properties. Synthesized compounds appeared to be moderate inhibitors of lipoxygenase, showing average values of ca. 40–60%. The stability of compounds at different pH levels was investigated, and it was shown that the ligand alone decomposes after approximately 1 h of experimentation, while complexes on its base possess noticeable stability, not completely decomposing even after 20 h. Cytotoxicity of compounds was studied by MTT-test. Paradox results were discovered, i.e., the addition of organotin fragment did not affect the cytostatic activity of the ligand and even lowered it in certain cases. These results show that a specific pharmaceutical target exists for the ligand alone and it is yet to be found. Luminescence was demonstrated for all the organotin complexes. It was shown, that the compounds emit in both powders and solutions with quantum yields of up to 67% in 100 µM DMSO solutions. The synergy of cytotoxic and luminescent properties lets us propose synthesized compounds as perspective theranostic agents for bioimaging.

## Figures and Tables

**Table 1:** Results of in vitro antioxidant screening.

Compound	Quantity of Reduced DPPH after 20 h, %	I (NBT-Test), %	I (LOX Inhibition Assay), %
**1**	- *	0.5 ± 0.1	51.7 ± 4.2
**2**	35.2 ± 1.9	6.3 ± 1.8	57.1 ± 4.7
**3**	41.3 ± 2.1	7.3 ± 2.0	61.1 ± 5.1
**4**	50.5 ± 2.8	−4.6 ± 1.2	47.5 ± 3.8
**5**	26.5 ± 1.7	5.9 ± 1.5	61.4 ± 5.3
**6**	47.4 ± 2.7	−28.0 ± 3.6	44.9 ± 3.7
**7**	- *	−40.5 ± 4.2	66.0 ± 5.9

* evaluated in a different way.

**Table 2:** Values established during the study of antiglycation activity.

Compound	Concentration, µM	Activity, %, M ± SEM	IC_50_, µM
**1**	1000	70.2 ± 8.0	2.8
	300	73.2 ± 1.9	
	100	61.6 ± 0.5	
	30	62.0 ± 0.7	
	10	60.1 ± 1.1	
	3	53.9 ± 2.5	
	1	0.6 ± 2.3	
	0.3	−1.9 ± 1.4	
Aminoguanidine	10,000	62.0 ± 2.0	5166.5
	3000	39.5 ± 2.2	
	1000	21.6 ± 1.8	

**Table 3:** Results of MTT-test for synthesized compounds.

Compound	IC_50_, µM			
	HCT116	MCF-7	A549	WI-38
**1**	1.7 ± 0.4	4.7 ± 0.8	2.2 ± 0.4	6.6 ± 1.2
**2**	2.4 ± 0.8	5.2 ± 1.0	2.2 ± 0.7	9.5 ± 3.1
**3**	1.4 ± 0.5	3.7 ± 0.5	1.5 ± 0.5	5.8 ± 1.5
**4**	10.0 ± 2.3	1.01 ± 0.4	14.5 ± 4.5	10.8 ± 3.5
**5**	34.3 ± 14.0	50.4 ± 12.6	43 ± 10.0	26.3 ± 11.3
**6**	20.0 ± 11.0	26.3 ± 3.5	20.2 ± 4.0	52.6 ± 14.6
**7**	20.5 ± 6.4	32.7 ± 9.5	31.9 ± 5.7	10.6 ± 3.5
Cisplatin	8.3 ± 3.5	15.5 ± 3.0	10.4 ± 1.5	16.3 ± 1.9

HCT-116—human colorectal carcinoma, MCF-7—human mammary adenocarcinoma, A549—human lung adenocarcinoma, WI-38—human fetal lung fibroblast.

**Table 4:** Photophysical properties of compounds **2**–**7**.

Compound	Quantum Yield, %				ε, (λ_max_, nm), M^−1^·cm^−1^	ε, (λ = 365 nm), M^−1^·cm^−1^	Luminosity (λ_max_), M^−1^·cm^−1^	Luminosity (λ = 365 nm), M^−1^·cm^−1^
	Powders *	DMSO Solution						
		1000 µM	100 µM	10 µM				
**2**	14 ± 2	39 ± 3	56 ± 1	17 ± 3	13,070, (403)	8617	771,130, (403)	508,403
**3**	14 ± 2	30 ± 2	45 ± 1	17 ± 2	14,020, (410)	4489	658,940, (410)	210,983
**4** *	4 ± 3	36 ± 3	66 ± 2	41 ± 3	5950, (409)	2181	398,650, (409)	146,127
**5**	8 ± 1	29 ± 2	37 ± 1	20 ± 2	9920, (412)	3080	664,640, (412)	206,360
**6**	6 ± 2	26 ± 2	67 ± 2	25 ± 2	13,450, (393)	9010	497,650, (393)	333,370
**7**	20 ± 2	32 ± 2	42 ± 1	21 ± 2	13,270, (402)	7002	557,340, (402)	294,084

* compound **4** is an oil.

## Data Availability

Copies of NMR, IR, and stability research spectra of the synthesized compounds are presented in the Supplementary Materials.

## Supplementary Materials

The following supporting information can be downloaded at: https://www.mdpi.com/article/10.3390/molecules27238359/s1, Figures show NMR, IR and stability study spectra of synthesized compounds.

## Sample Availability

Samples of the compounds are not available from the authors.
