# Supplementary material for: Cytotoxic and Luminescent Properties of Novel Organotin Complexes with Chelating Antioxidant Ligand
Source: Molecules. 2022 Nov 30;27(23):8359. doi: 10.3390/molecules27238359 (PMC9741287; doi:10.3390/molecules27238359)
Supplement: Supplementary file 1 [file molecules-27-08359-s001.zip › molecules-2035192-supplementary.pdf]

Cytotoxic and luminescent properties of novel organotin complexes with chelating antioxidant ligand

Evgeny Nikitin [1], Ekaterina Mironova [1], Dmitry Shpakovsky [1], Yulia Gracheva [1], Daniil Koshelev [1], Valentina Utochnikova [1], Konstantin Lyssenko [1], Yury Oprunenko [1], Dmitry Yakovlev [2], Roman Litvinov [2], Mariya Seryogina [2], Alexander Spasov [2] and Elena Milaeva [1]*

Supporting information

Compound **1**

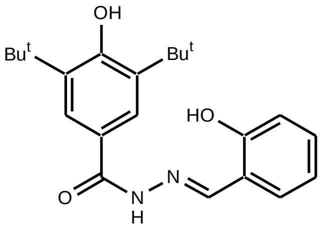

Scheme S1. Structural formula of compound **1**.

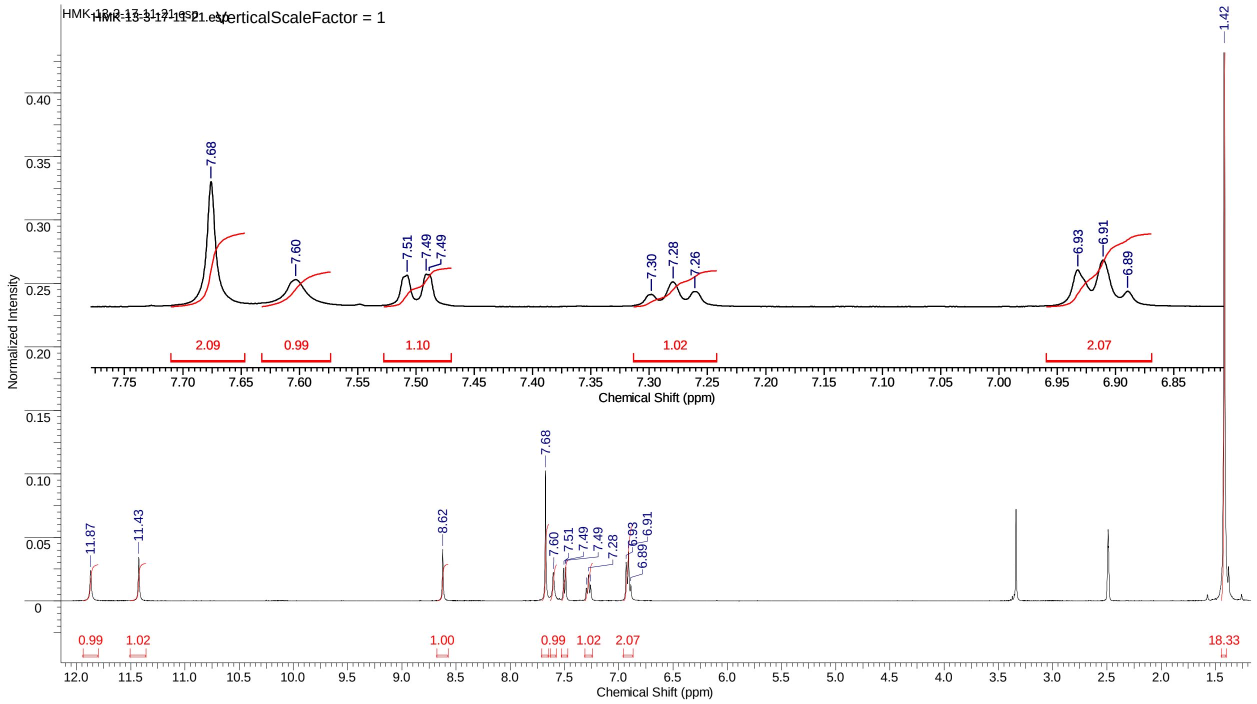

Figure S1. ^{1}H NMR spectrum of compound **1**.

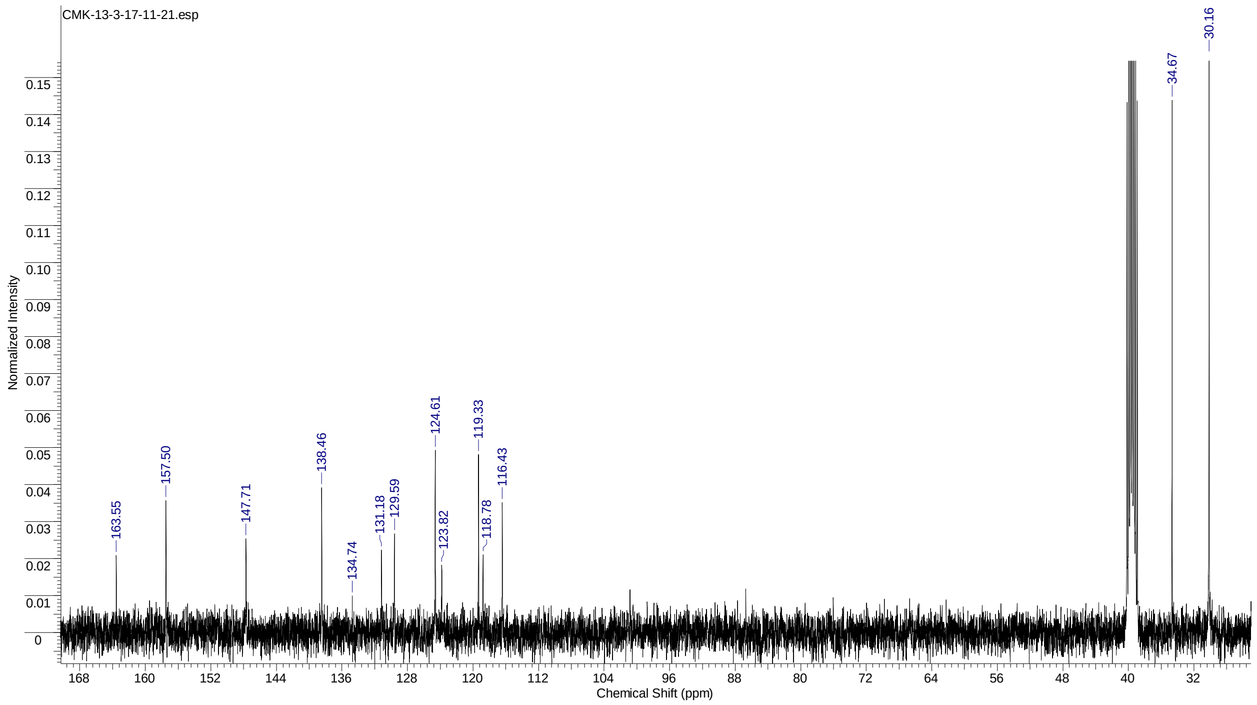

Figure S2. ^{13}C NMR spectrum of compound **1**.

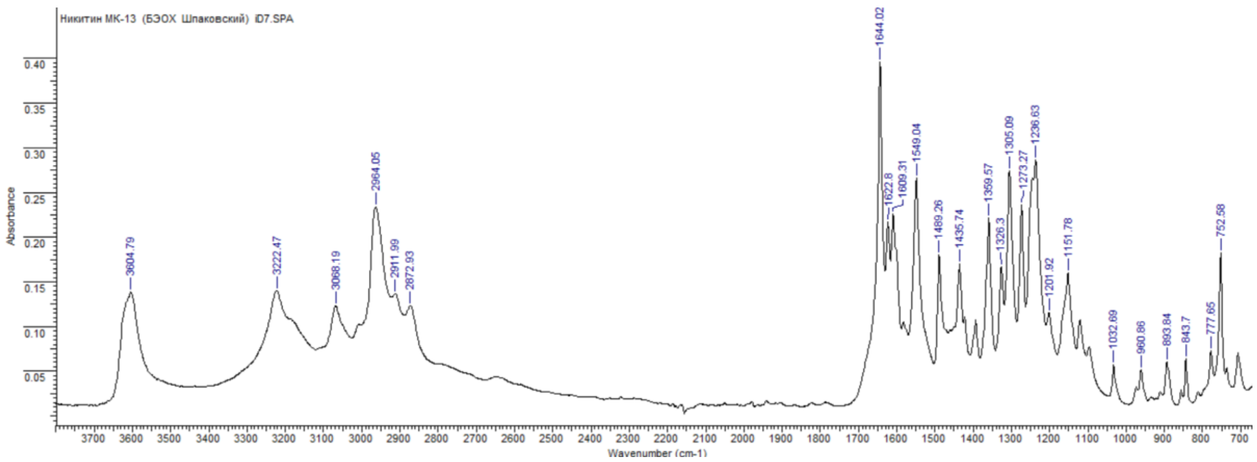

Figure S3. IR spectrum of compound **1**.

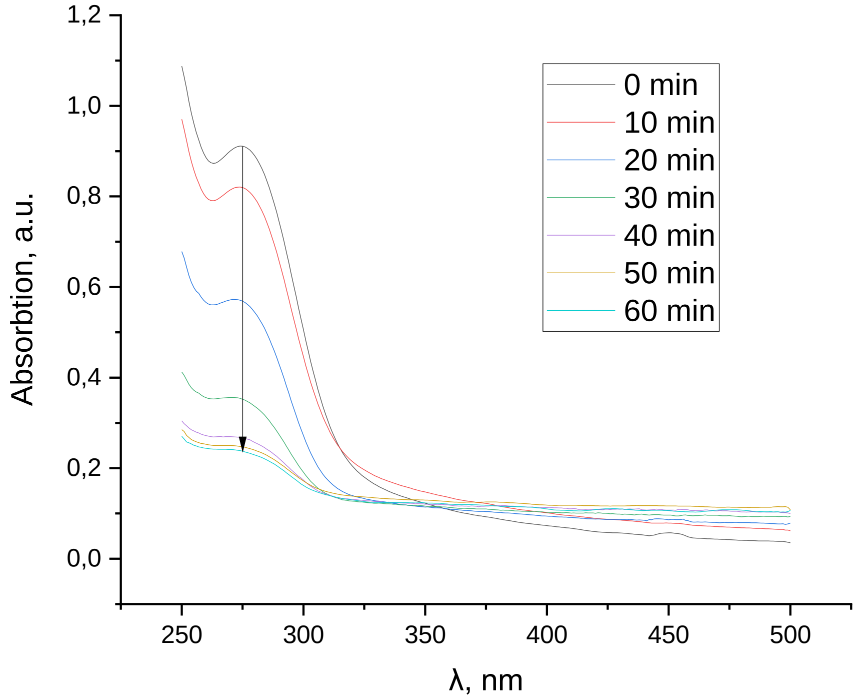

Figure S4. Hydrolysis of compound **1** for 1 hour at pH=5.

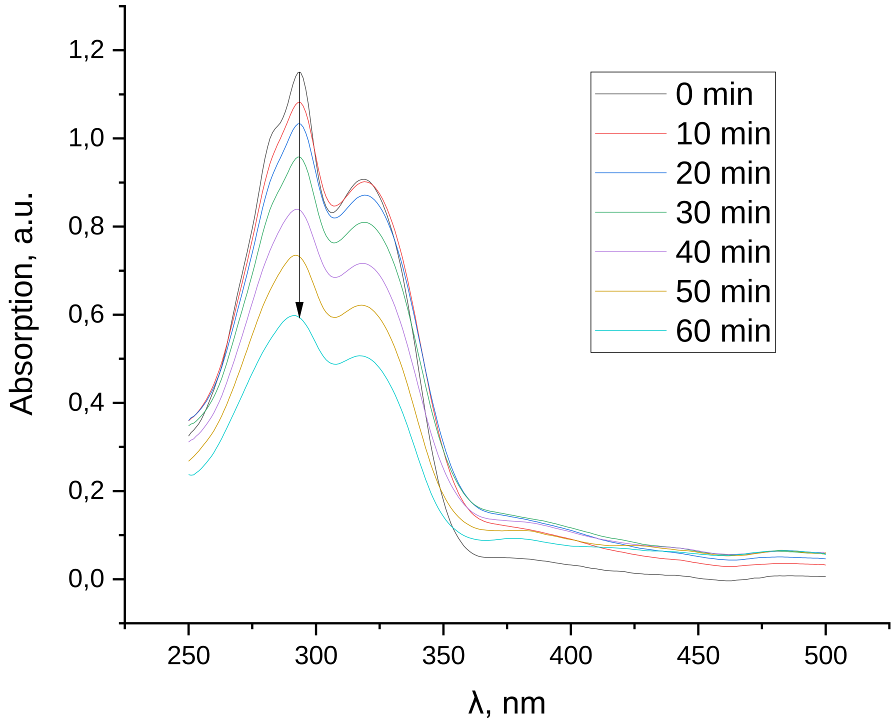

Figure S5. Hydrolysis of compound **1** for 1 hour at pH=7.

Compound **2**

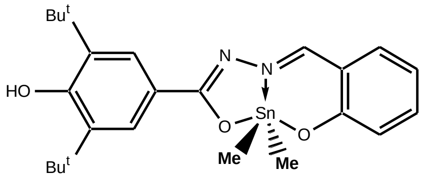

Scheme S2. Structural formula of compound **2**.

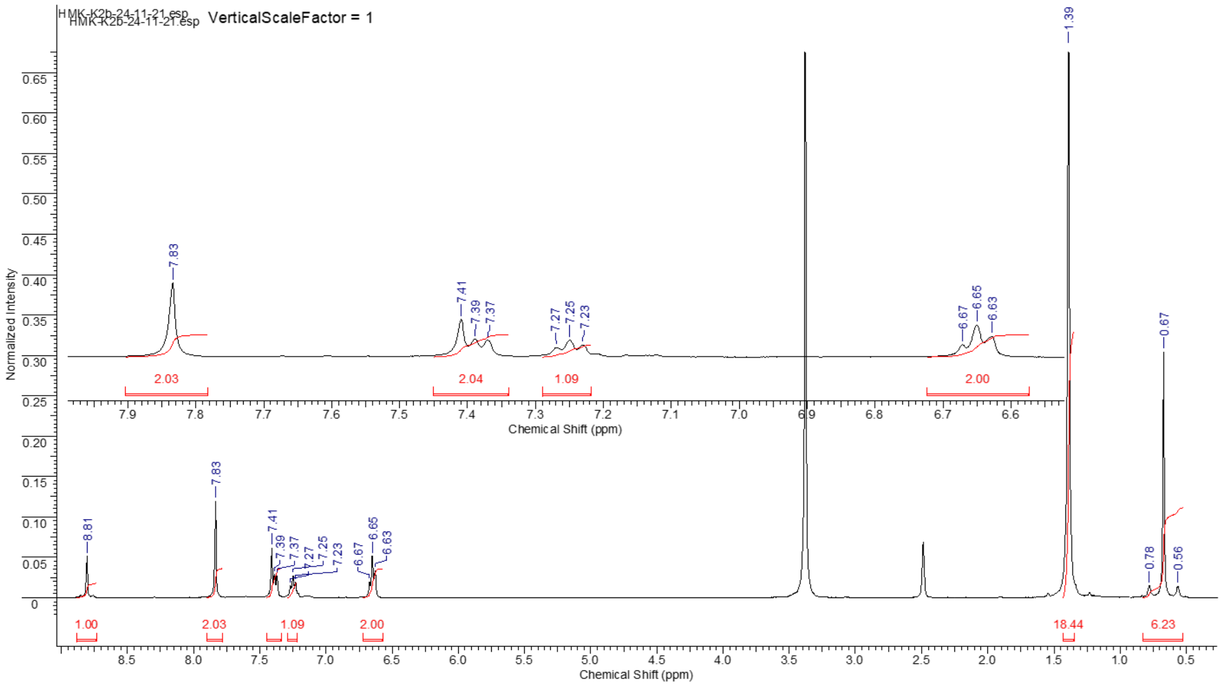

Figure S6. ^{1}H NMR spectrum of compound **2**.

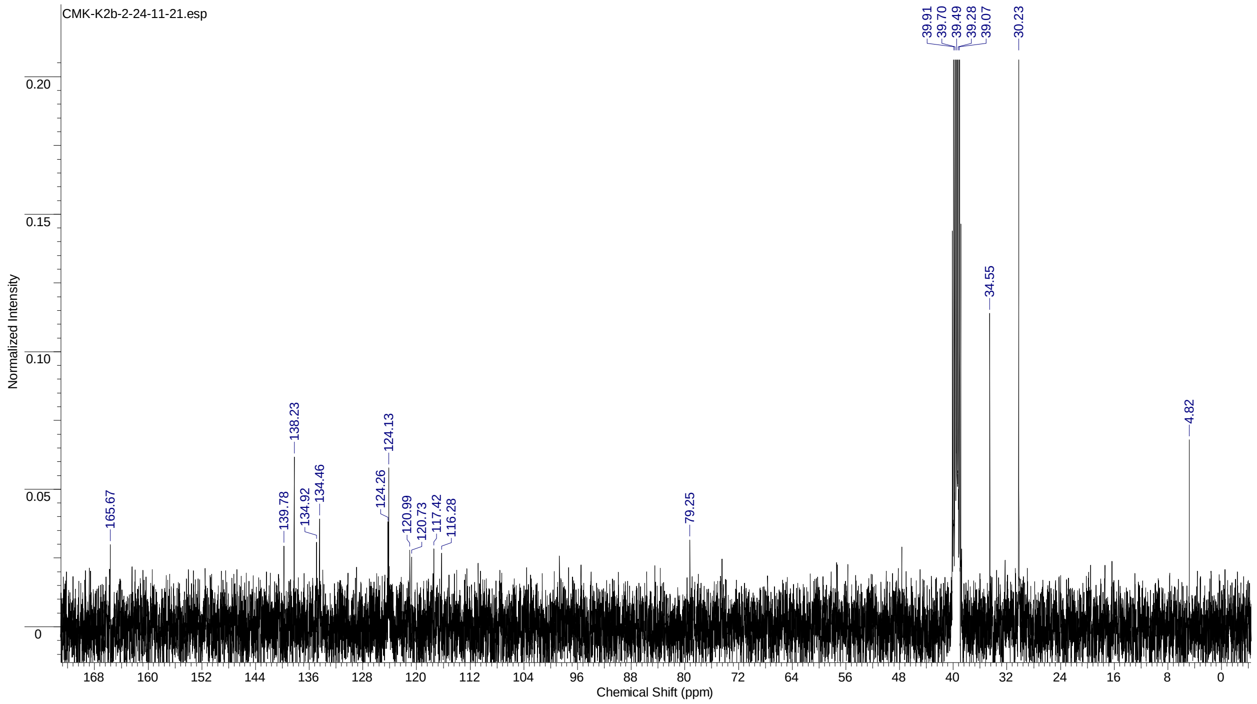

Figure S7. ^{13}C NMR spectrum of compound **2**.

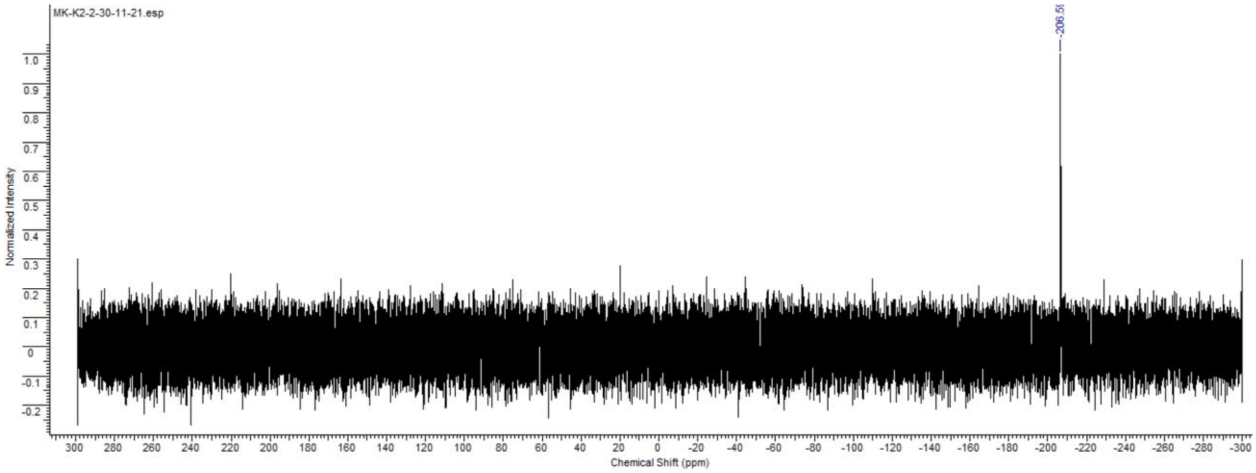

Figure S8. ^{119}Sn NMR spectrum of compound **2**.

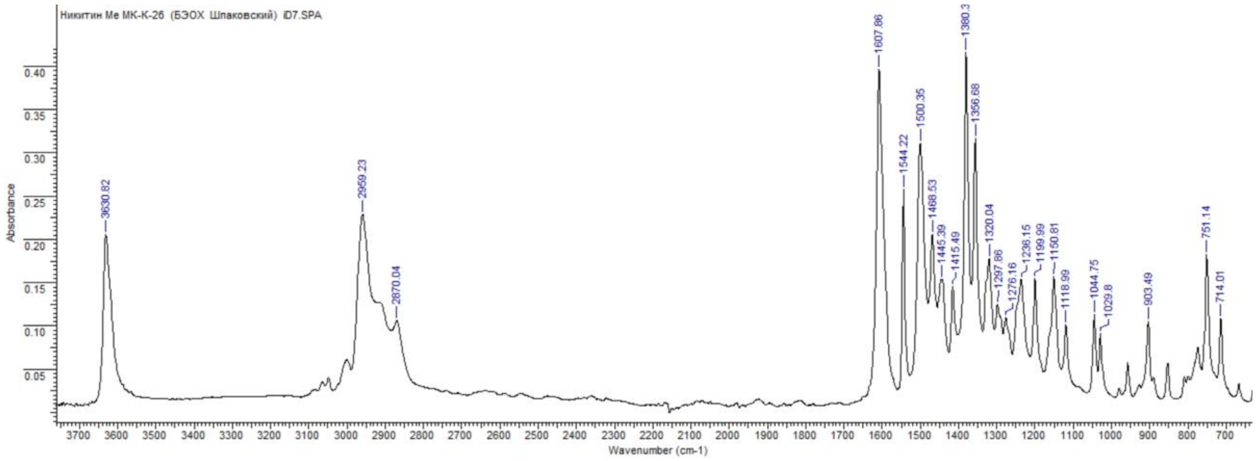

Figure S9. IR spectrum of compound **2**.

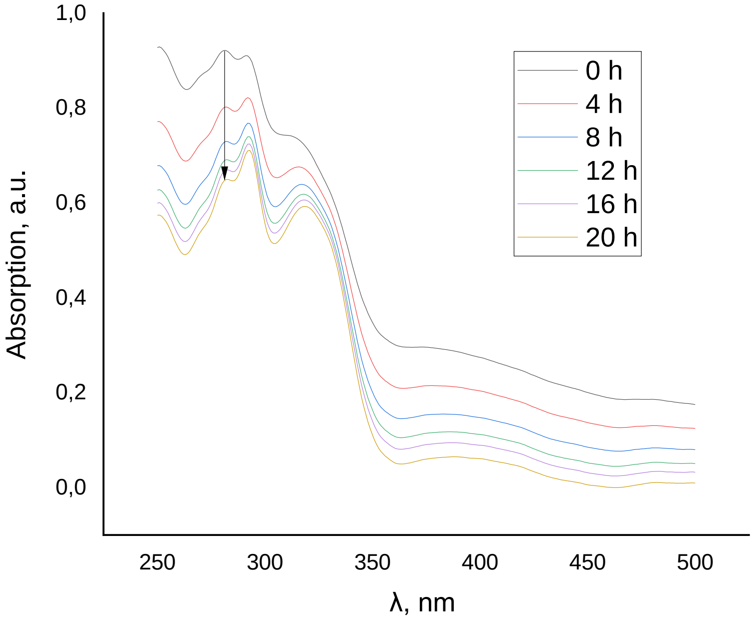

Figure S10. Hydrolysis of compound **2** for 20 hours at pH=5.

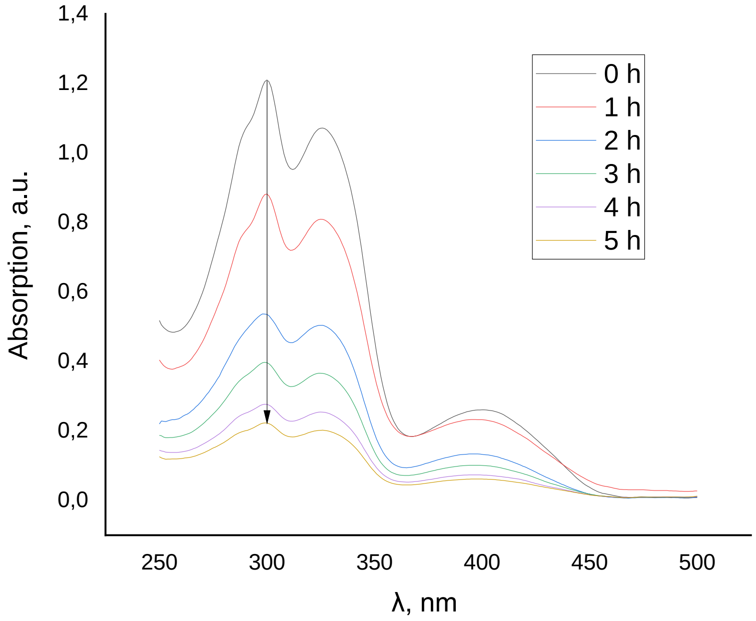

Figure S11. Hydrolysis of compound **2** for 5 hours at pH=7.

Compound 3

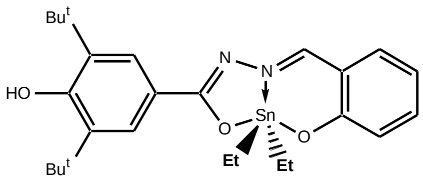

Scheme S3. Structural formula of compound **3**.

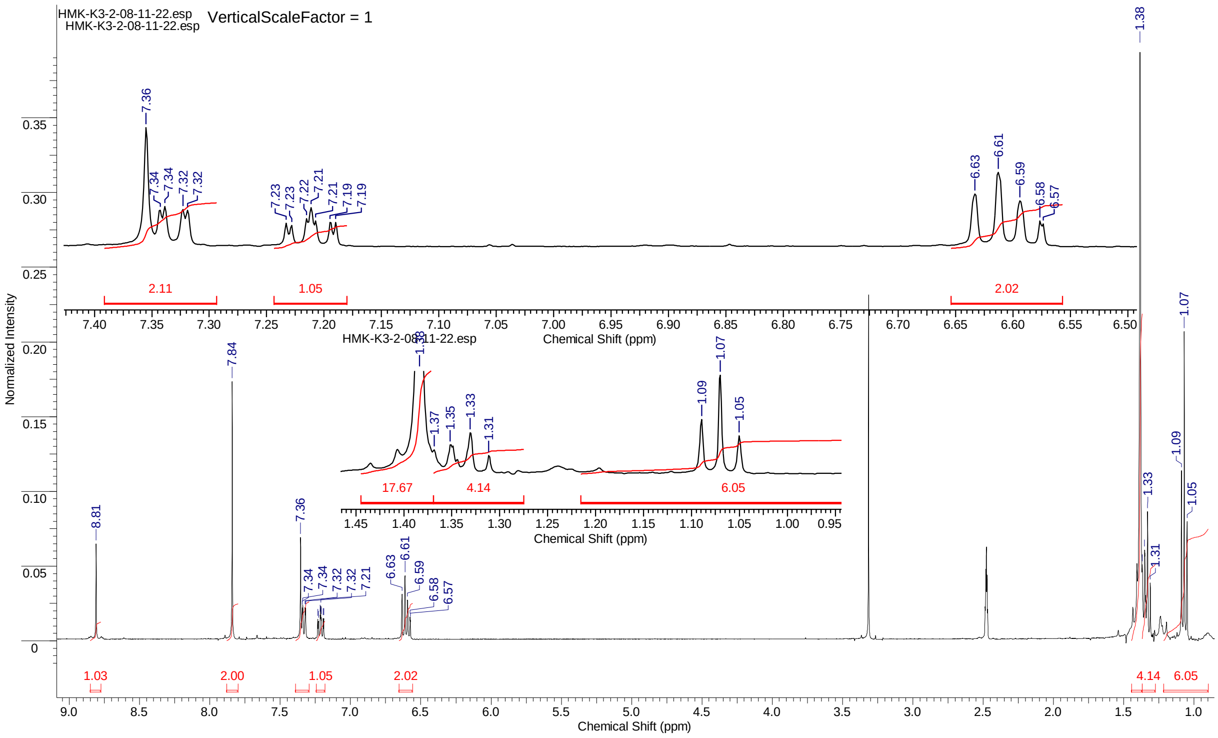

Figure S12. ^{1}H NMR spectrum of compound **3**.

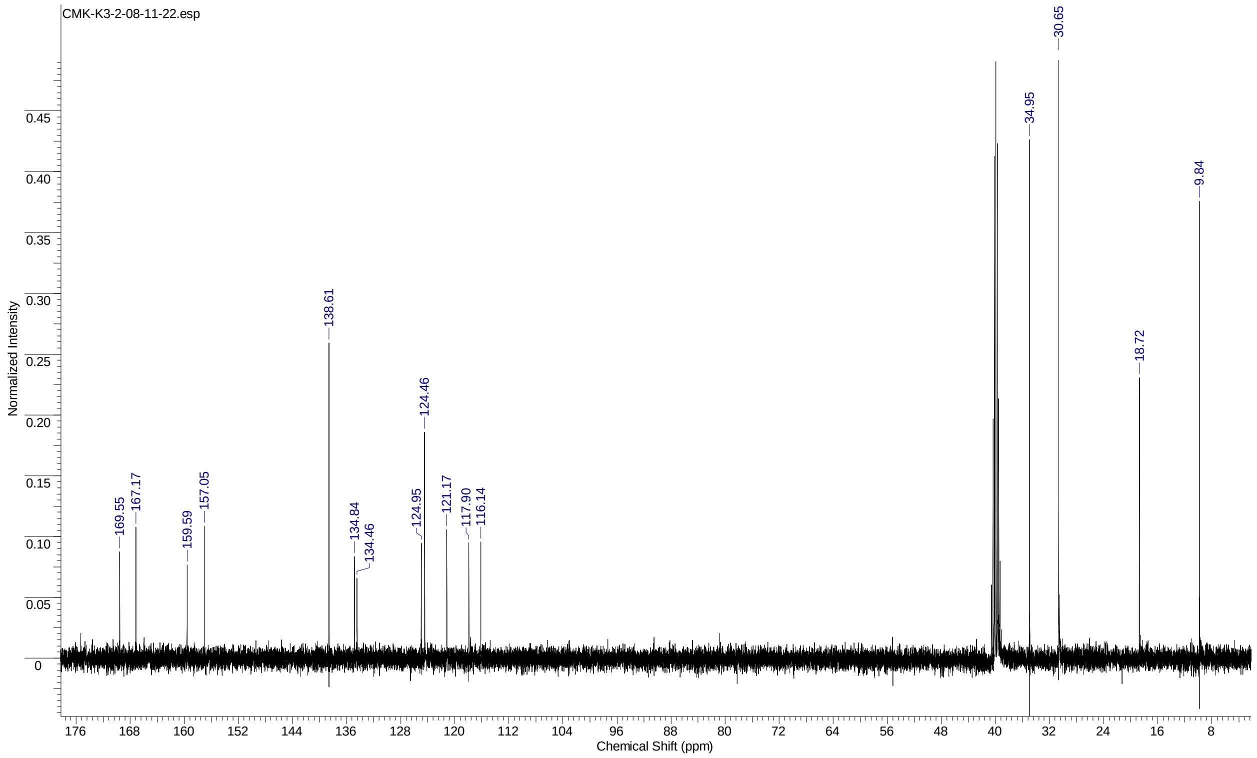

Figure S13. ^{13}C NMR spectrum of compound **3**.

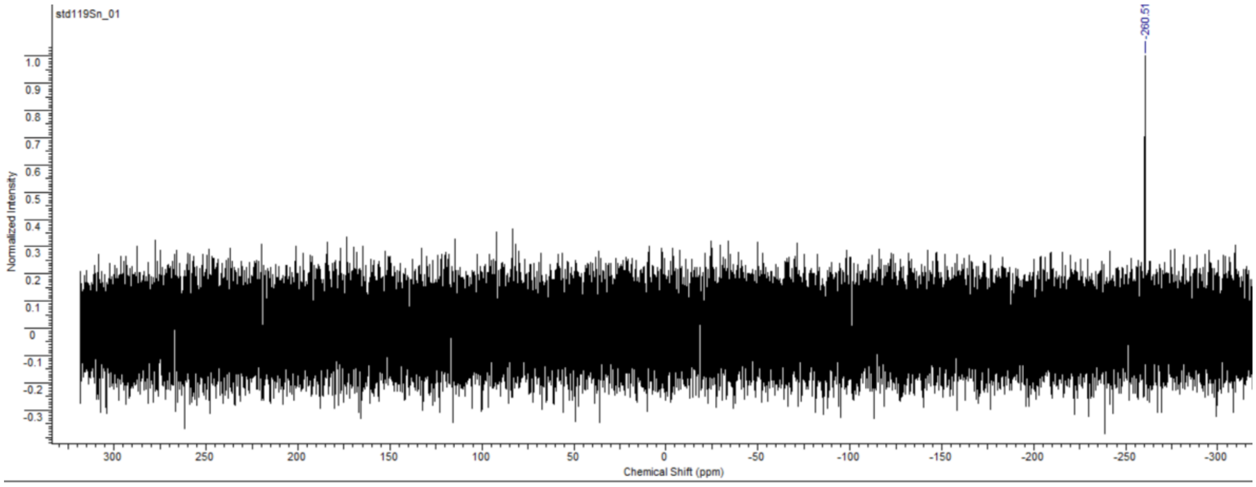

Figure S14. ^{119}Sn NMR spectrum of compound **3**.

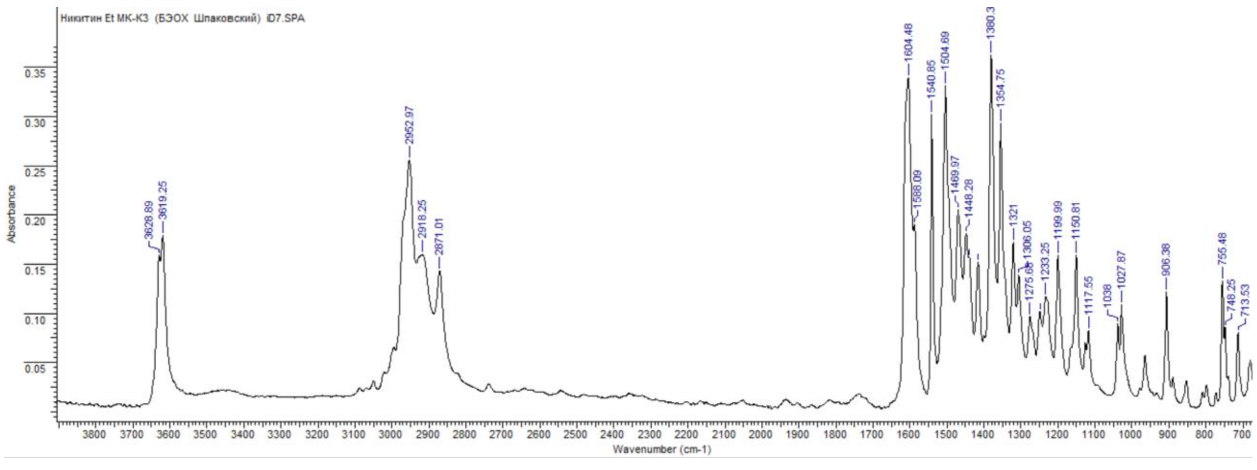

Figure S15. IR spectrum of compound **3**.

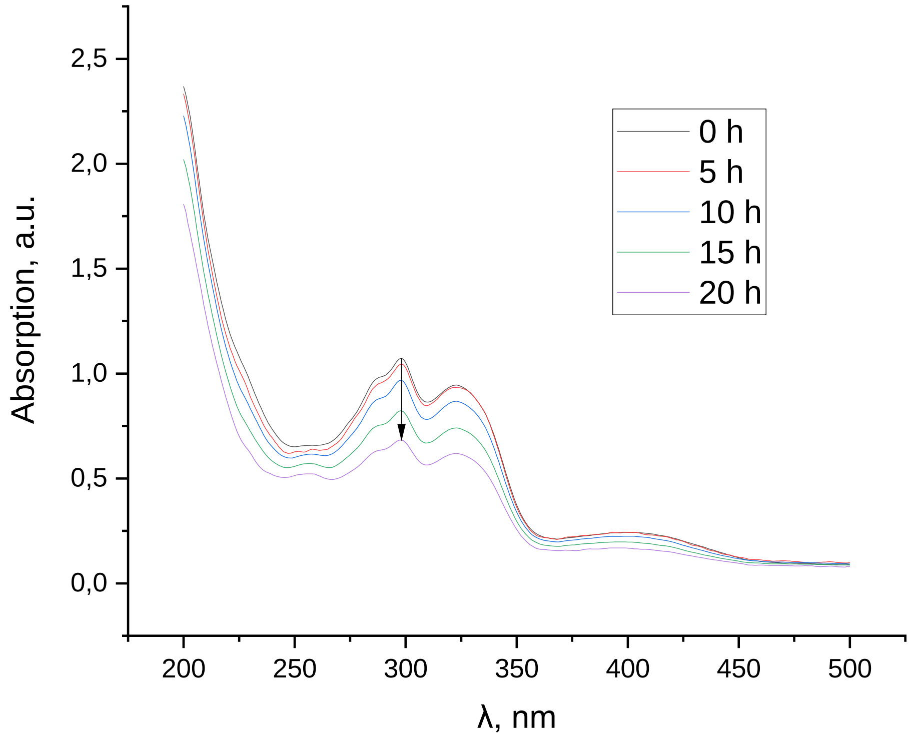

Figure S16. Hydrolysis of compound **3** for 20 hours at pH=5.

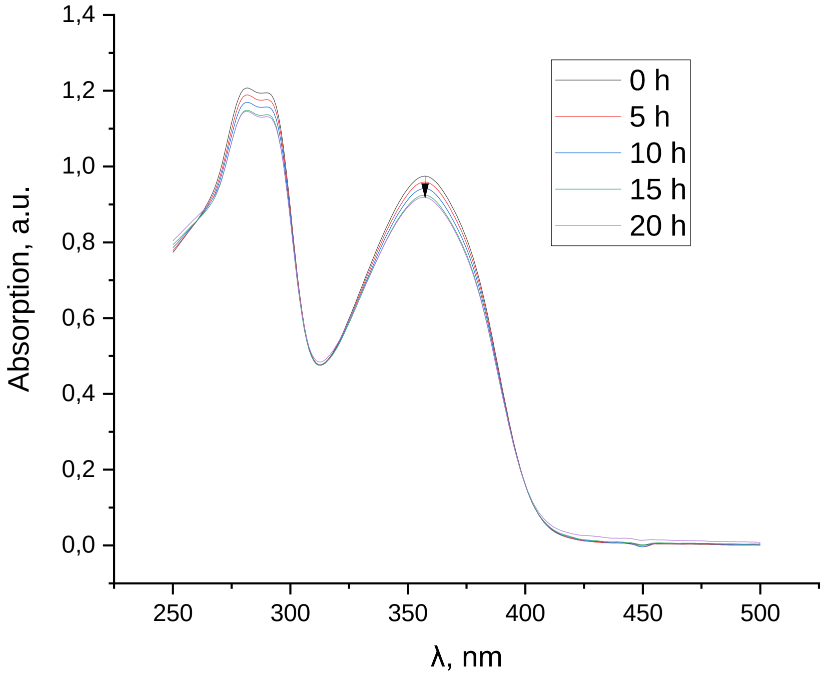

Figure S17. Hydrolysis of compound **3** for 20 hours at pH=7.

Compound 4

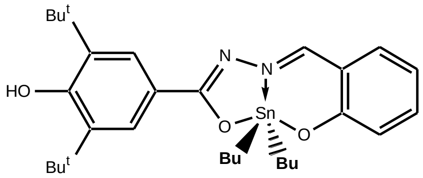

Scheme S4. Structural formula of compound **4**.

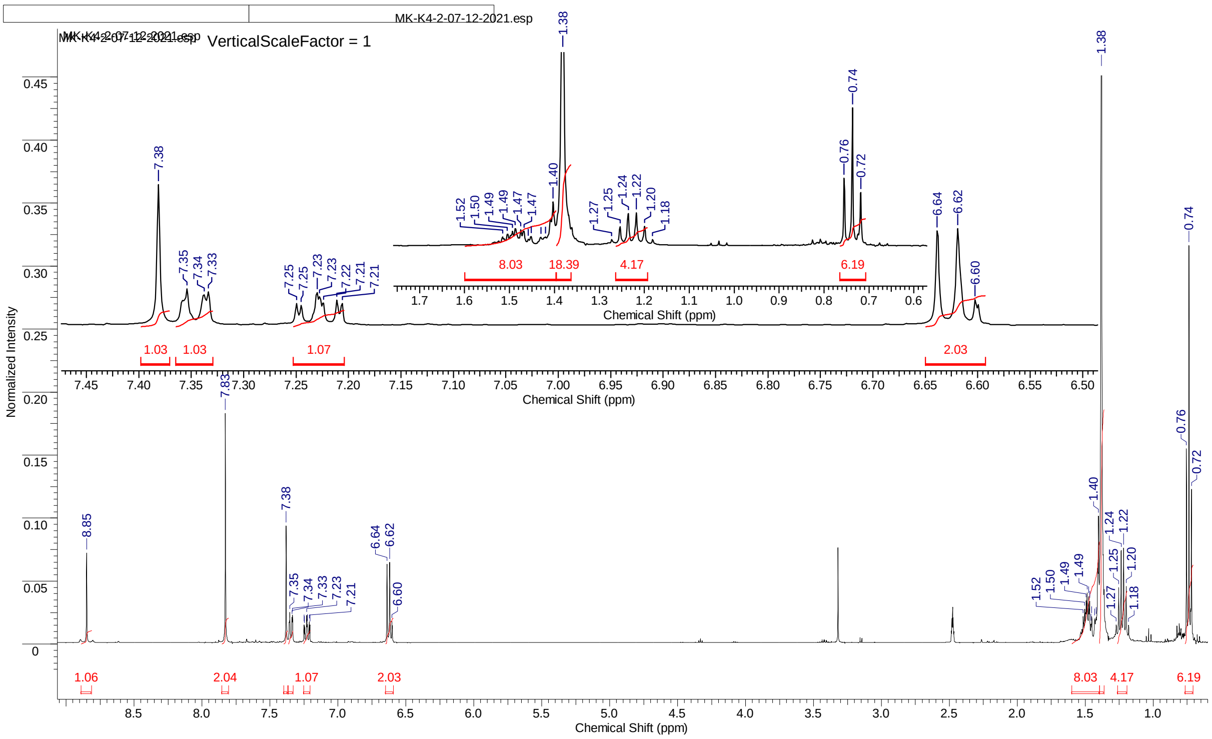

Figure S18. ^{1}H NMR spectrum of compound **4**.

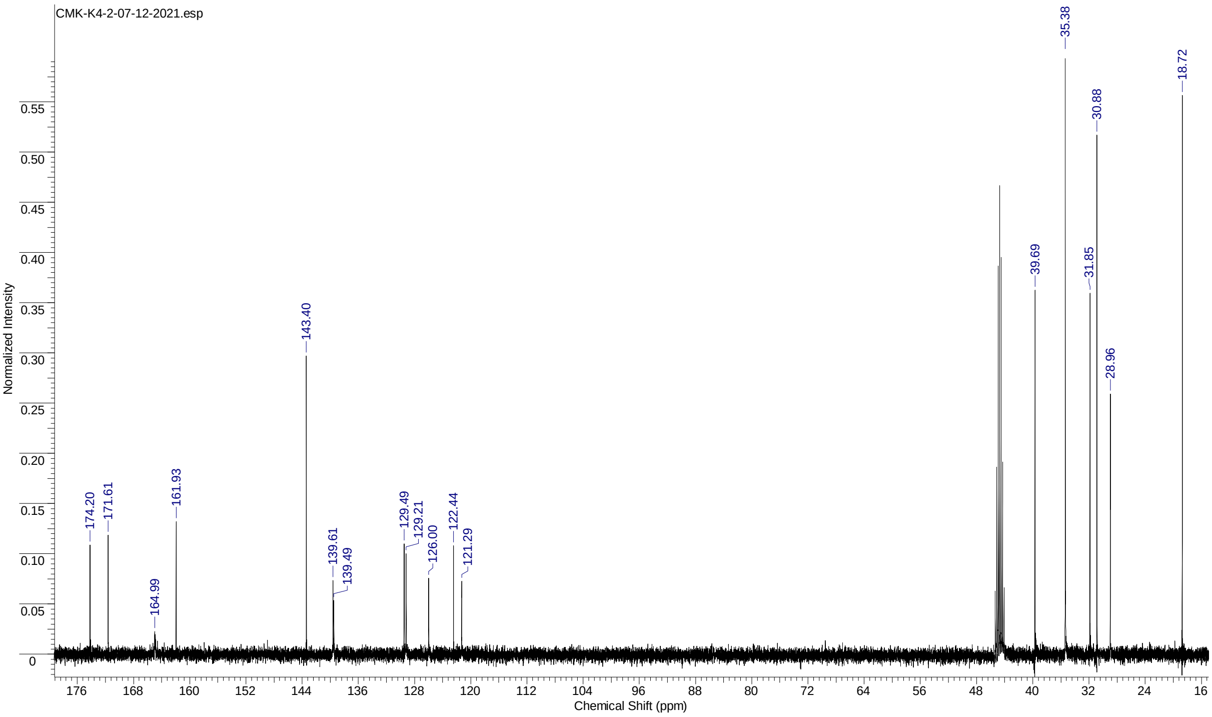

Figure S19. ^{13}C NMR spectrum of compound **4**.

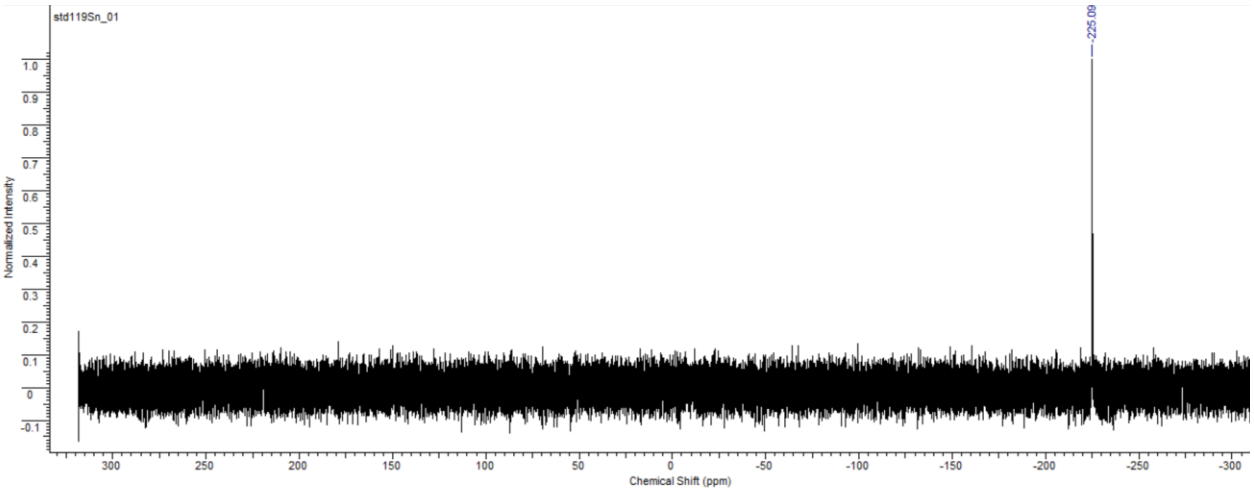

Figure S20. ^{119}Sn NMR spectrum of compound **4**.

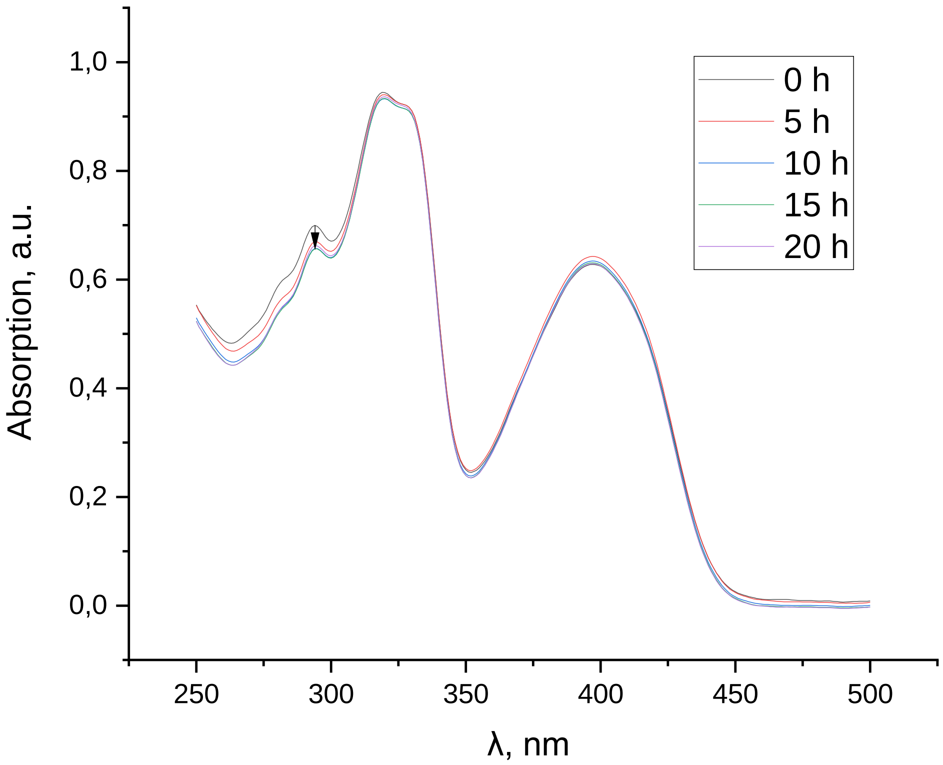

Figure S21. Hydrolysis of compound **4** for 20 hours at pH=5.

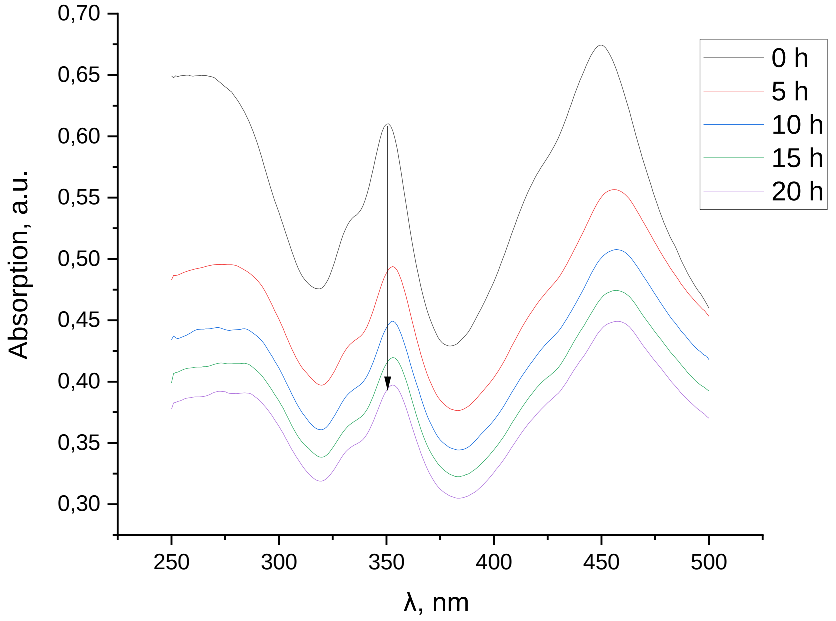

Figure S22. Hydrolysis of compound **4** for 20 hours at pH=7.

Compound **5**

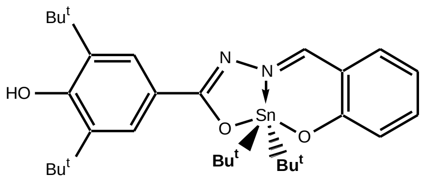

Scheme S5. Structural formula of compound **5**.

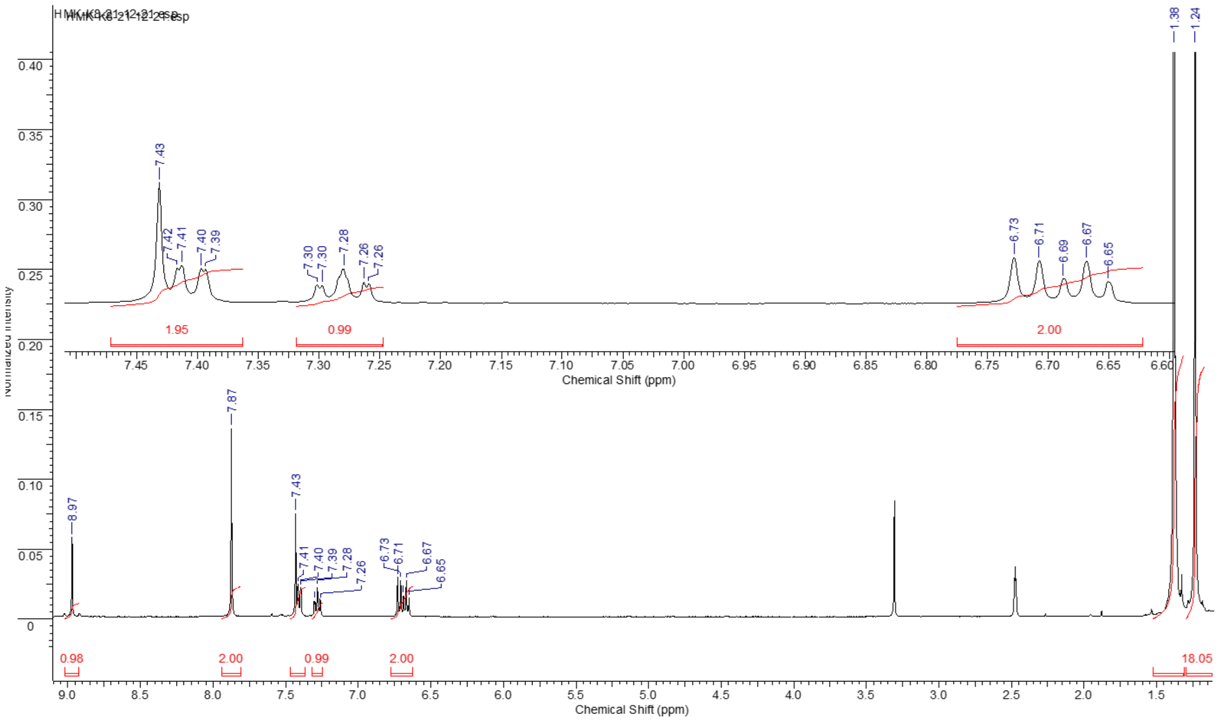

Figure S23. ^{1}H NMR spectrum of compound **5**.

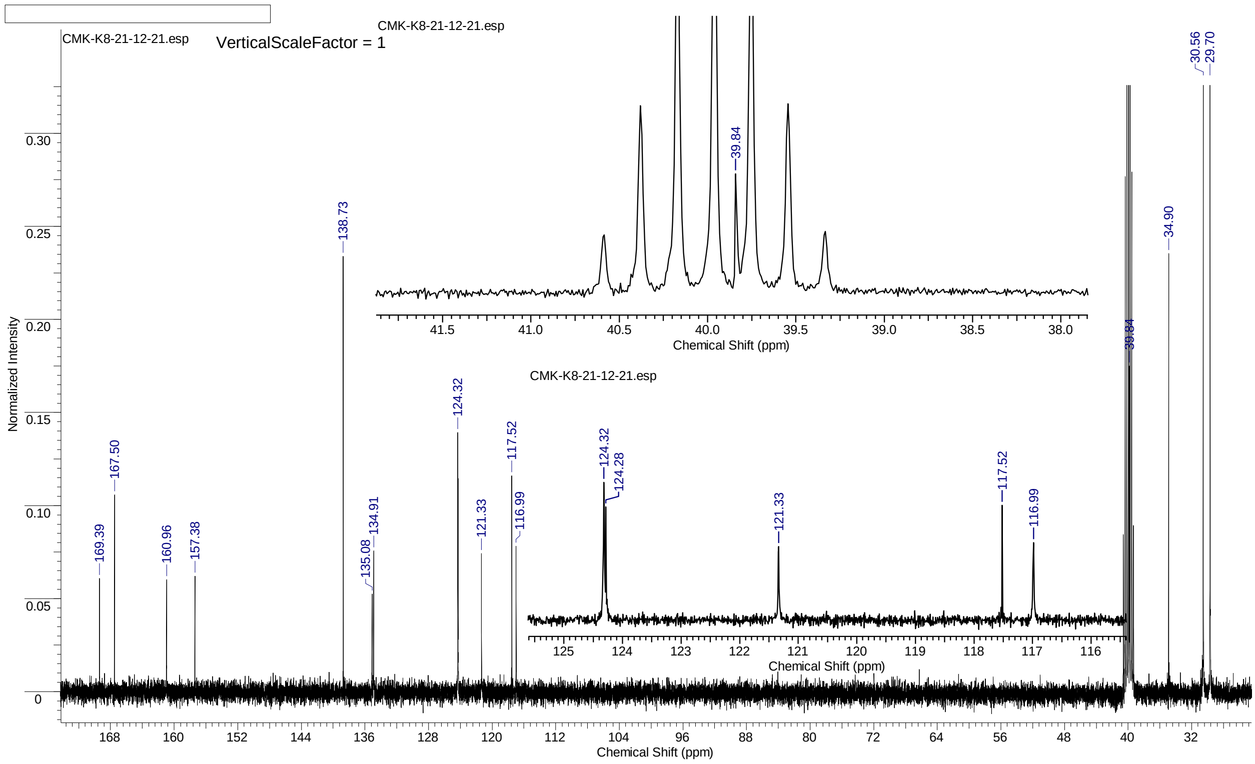

Figure S24. ^{13}C NMR spectrum of compound **5**.

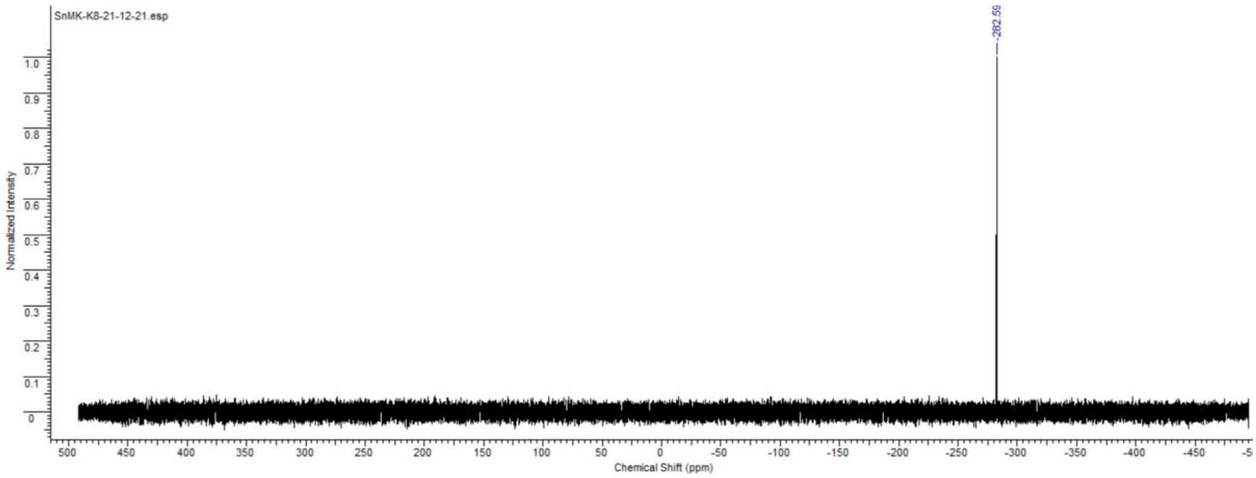

Figure S25. ^{119}Sn NMR spectrum of compound **5**.

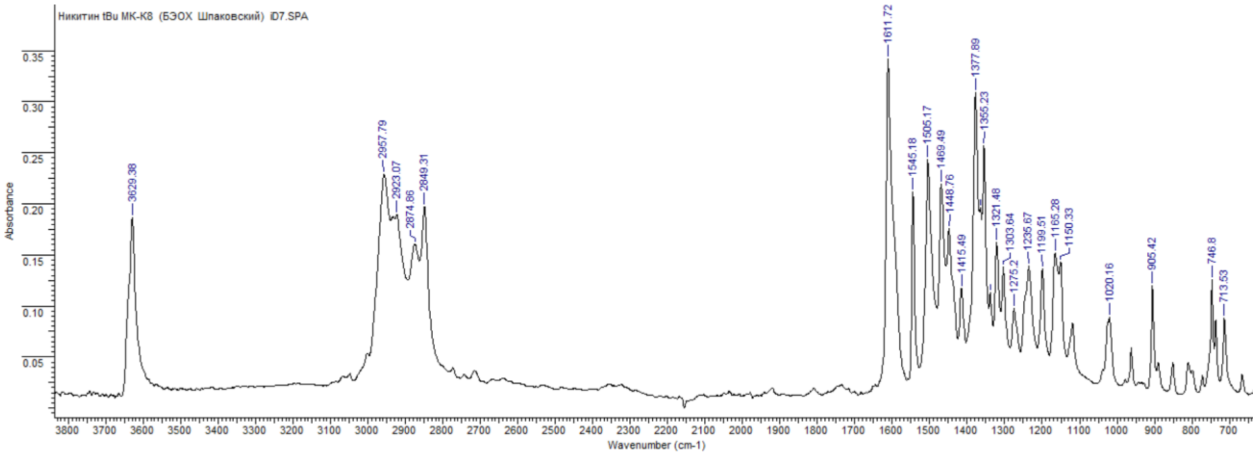

Figure S26. IR spectrum of compound **5**.

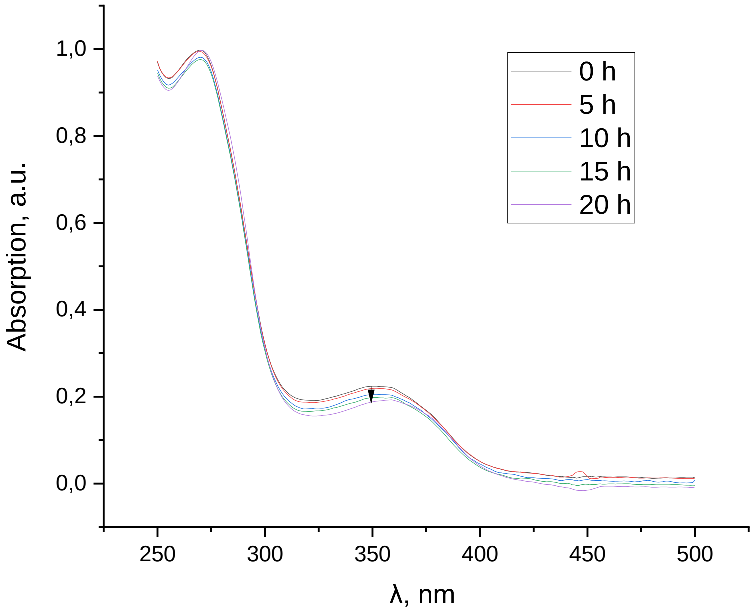

Figure S27. Hydrolysis of compound **5** for 20 hours at pH=5.

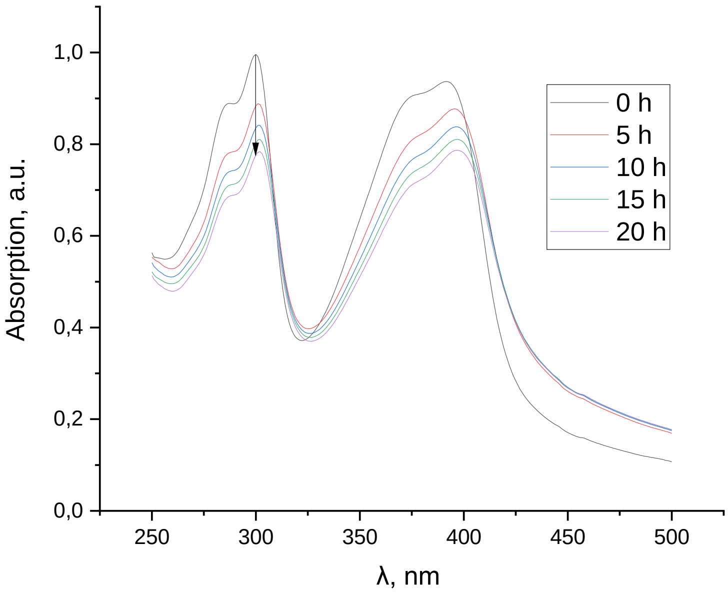

Figure S28. Hydrolysis of compound **5** for 20 hours at pH=7.

Compound **6**

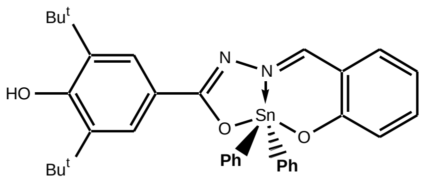

Scheme S6. Structural formula of compound **6**.

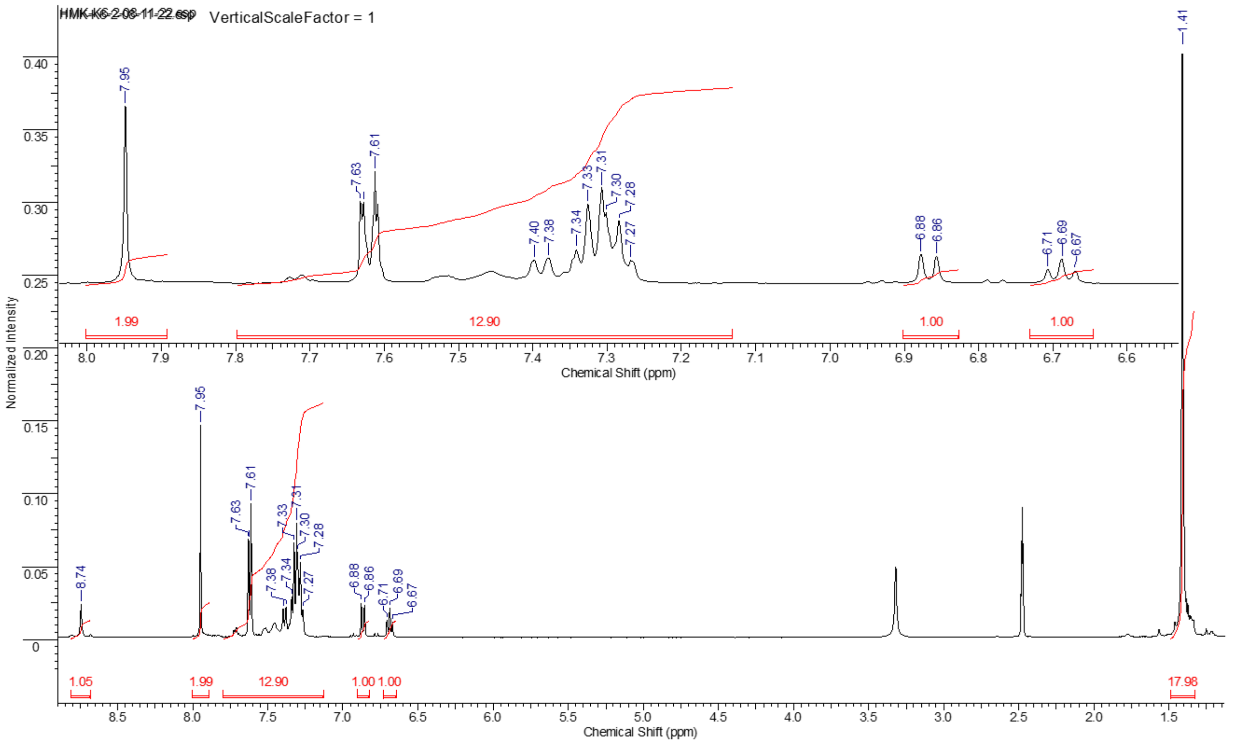

Figure S29. ^{1}H NMR spectrum of compound **6**.

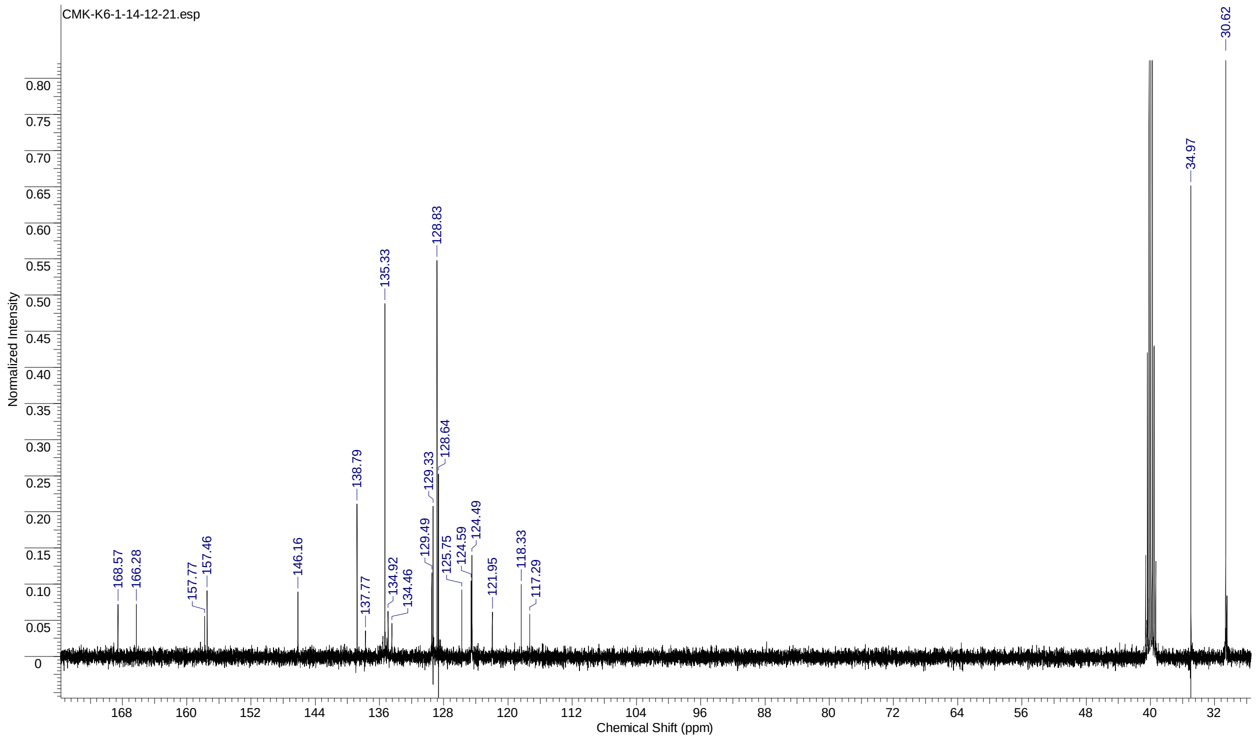

Figure S30. ^{13}C NMR spectrum of compound **6**.

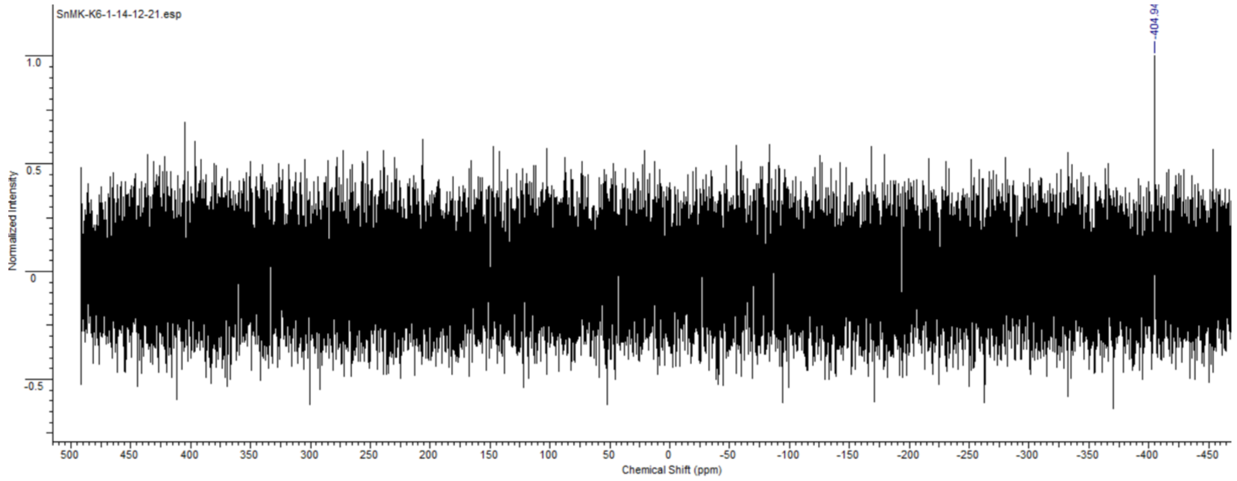

Figure S31. ^{119}Sn NMR spectrum of compound **6**.

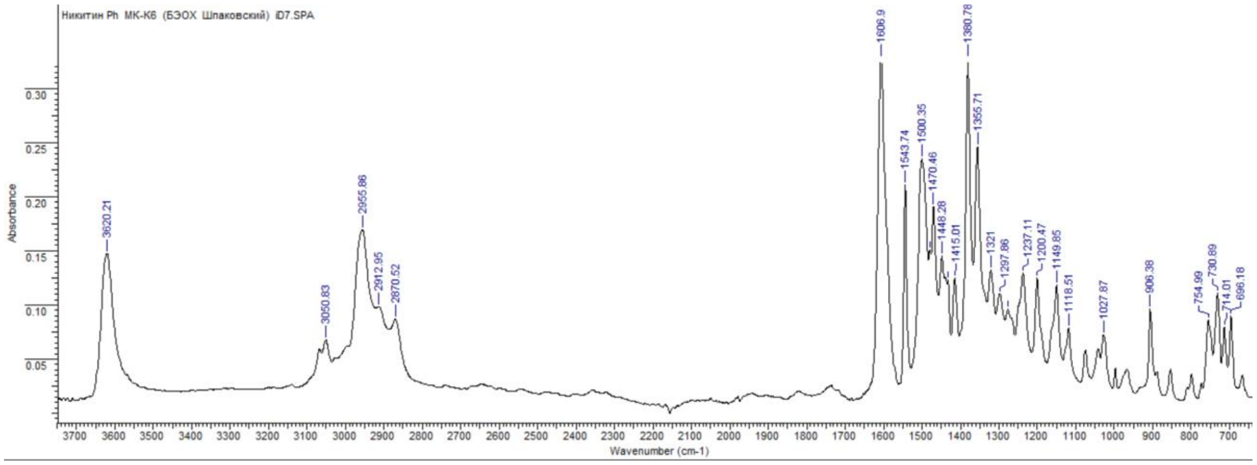

Figure S32. IR spectrum of compound **6**.

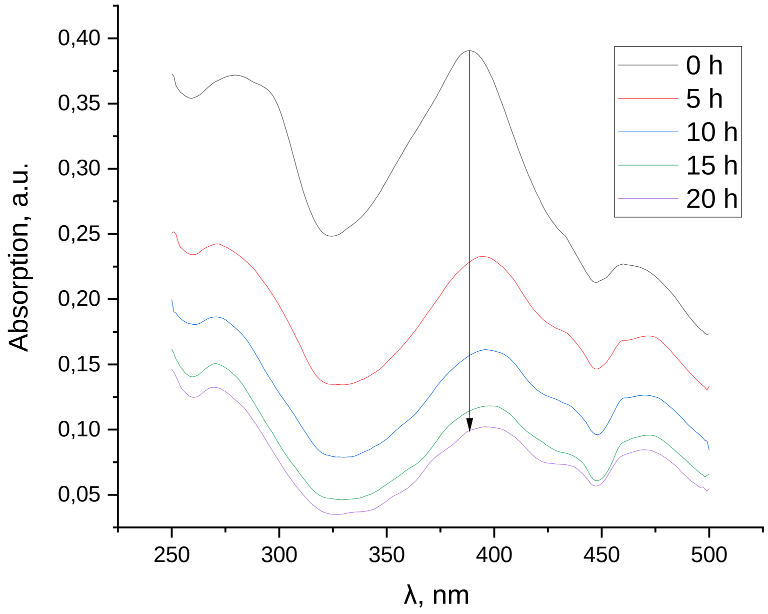

Figure S33. Hydrolysis of compound **6** for 20 hours at pH=5.

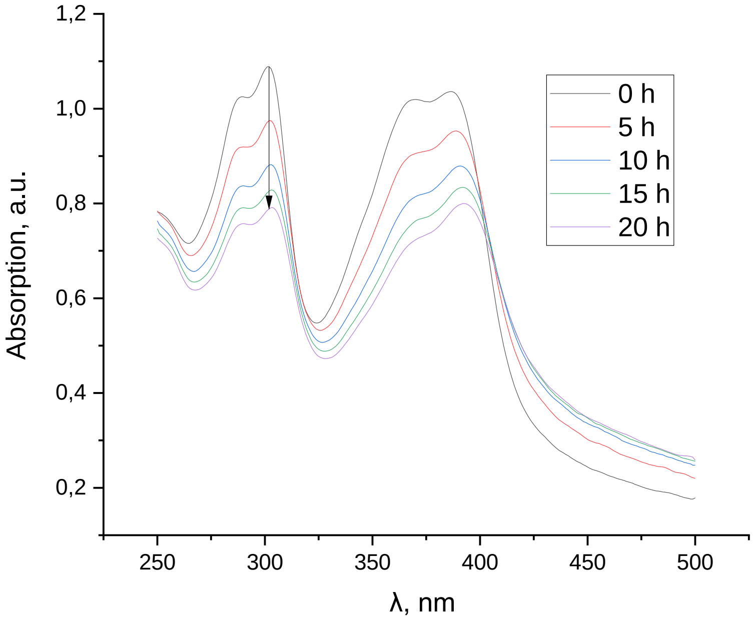

Figure S34. Hydrolysis of compound **6** for 20 hours at pH=7.

Compound **7**

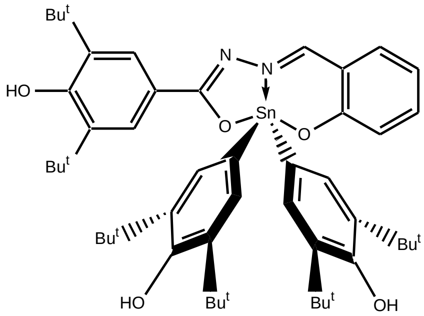

Scheme S7. Structural formula of compound **7**.

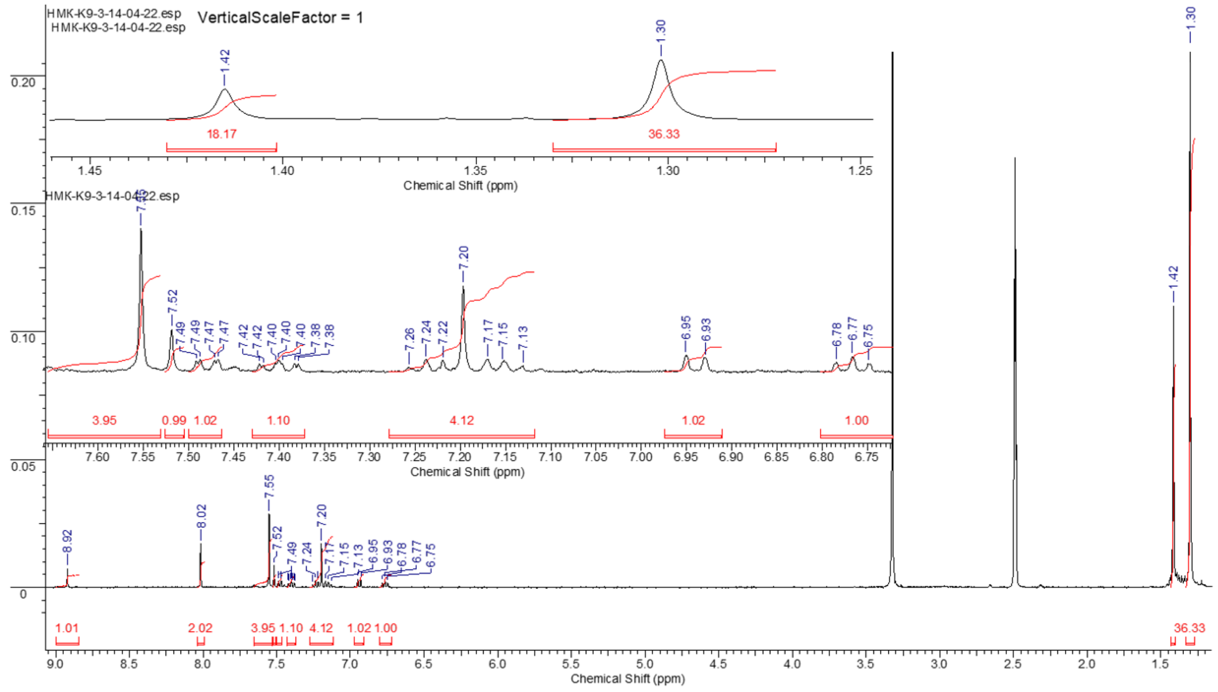

Figure S35. ^{1}H NMR spectrum of compound **7**.

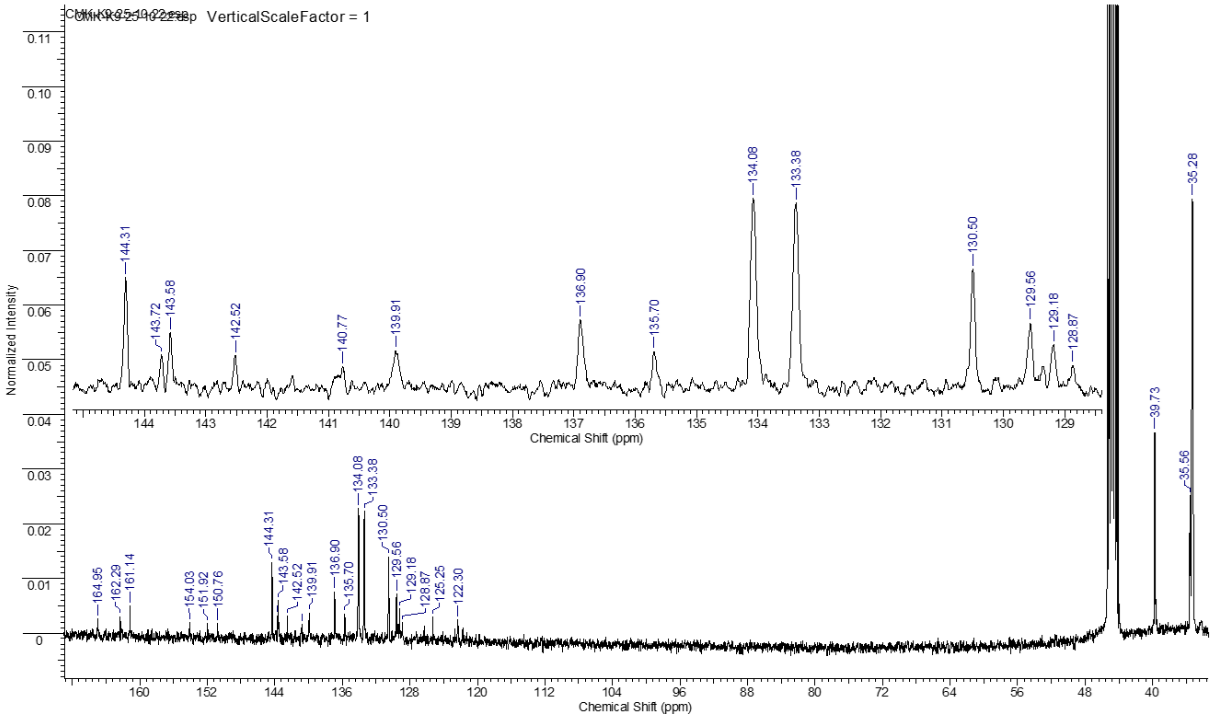

Figure S36. ^{13}C NMR spectrum of compound **7**.

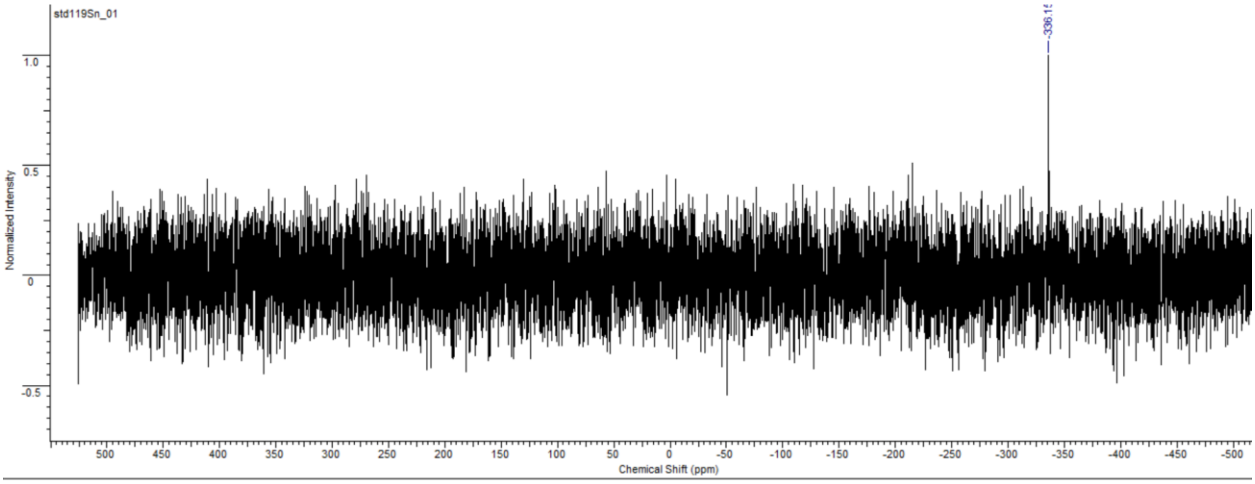

Figure S37. ^{119}Sn NMR spectrum of compound **7**.

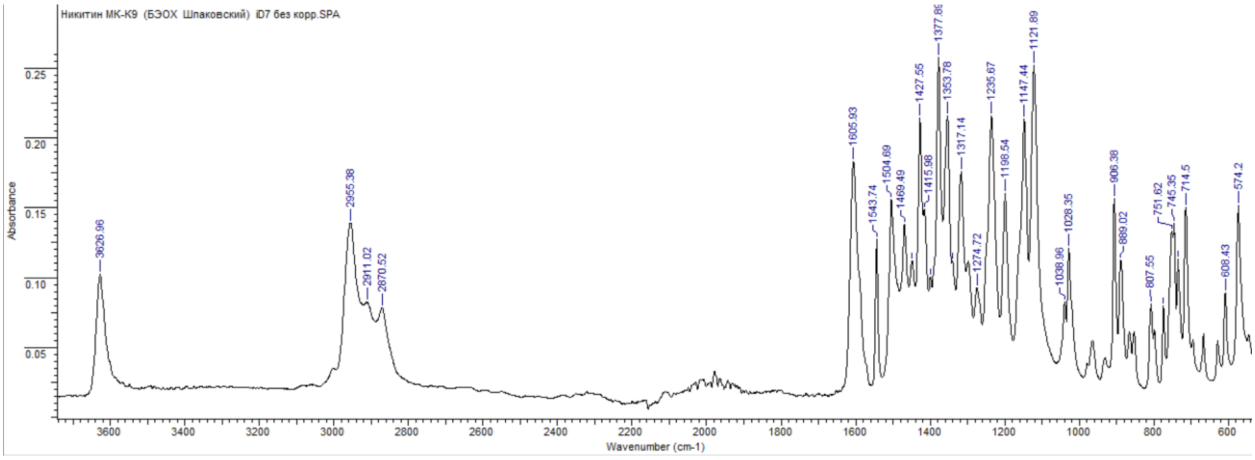

Figure S38. IR spectrum of compound **7**.

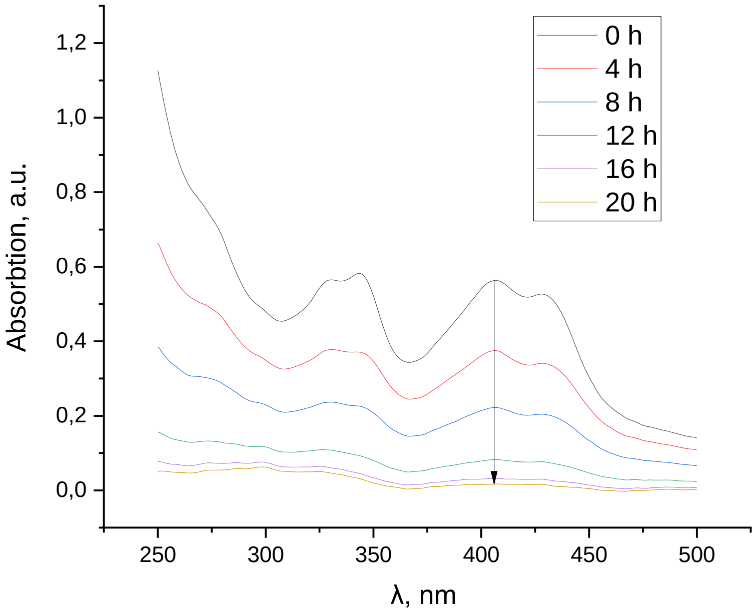

Figure S39. Hydrolysis of compound **7** for 20 hours at pH=5.

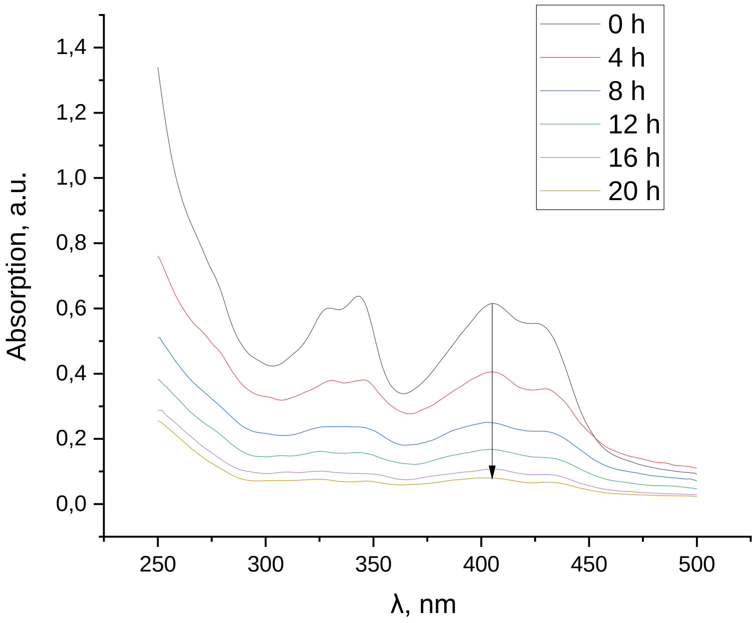

Figure S40. Hydrolysis of compound **7** for 20 hours at pH=7.
